# Reviewing Glycosyl‐Inositols: Natural Occurrence, Biological Roles, and Synthetic Techniques

**DOI:** 10.1002/cbic.202400823

**Published:** 2025-03-02

**Authors:** Alfonso Miranda‐Molina, Laura Alvarez, Mayra Antunez‐Mojica, Benjamín Velasco‐Bejarano

**Affiliations:** ^1^ Departamento de Ingeniería Celular y Biocatálisis Instituto de Biotecnología Universidad Nacional Autónoma de México. Av. Universidad 2001, Col. Chamilpa, C. P. 62210 Cuernavaca, Morelos México; ^2^ LANEM-Centro de Investigaciones Químicas-IICBA Universidad Autónoma del Estado de Morelos Avenida Universidad 1001 Cuernavaca Morelos 62209 Mexico; ^3^ cCentro de Investigaciones Químicas-IICBA Universidad Autónoma del Estado de Morelos Avenida Universidad 1001 Cuernavaca Morelos 62209 México; ^4^ CONAHCYT-Centro de Investigaciones Químicas-IICBA Universidad Autónoma del Estado de Morelos Avenida Universidad 1001 Cuernavaca Morelos 62209 México; ^5^ Sección de Química Orgánica Departamento de Ciencias Químicas Facultad de Estudios Superiores Cuautitlán-UNAM Av. 1 de Mayo S/N, Col. Sta. Ma. Las Torres Cuautitlán Izcalli 54740 Estado de México C.P.

**Keywords:** Inositols, Methyl-inositols, Glycosyl inositols, Chemical glycosylation, Enzymes, Glycosidases, Glycosyltransferases.

## Abstract

Glycosyl‐inositols are molecules consisting of one or more α‐ or β‐D‐glycosyl residues bonded primarily to inositol or methyl‐inositol. These derivatives are found in plants, yeast, bacteria, and parasites, and exhibit diverse biological properties. The limited availability of glycosyl inositols from natural sources has led to significant interest in chemical and enzymatic synthesis techniques due to their potential applications in various fields. This review provides a comprehensive overview of inositols, methyl‐inositols, and primarily glycosyl inositols, focusing on their classification, natural occurrence, biological roles, and potential applications across different disciplines. Inositols, particularly *myo*‐inositol and its derivatives are widely distributed in plants and play essential roles in biochemical processes and metabolic functions in different organs and tissues. Glycosyl inositols, including glycosylphosphatidylinositols, glycosyl inositol phosphorylceramides, phosphatidylinositol mannosides, monoglycosyl and diglycosyl derivatives, are discussed, emphasizing their structural diversity and biological functions. Methods for their chemical and enzymatic synthesis are also reviewed, highlighting recent advances and challenges in the field. Overall, this comprehensive review underscores the significance of glycosyl inositols as versatile molecules with diverse biological functions and promising applications in scientific research and industry.

## Graphical abstract

Overall, this comprehensive review underscores the significance of glycosyl inositols as versatile molecules with diverse biological functions and promising applications in scientific research and industry. Methods for their chemical and enzymatic synthesis are also reviewed, highlighting recent advances and challenges in the field.

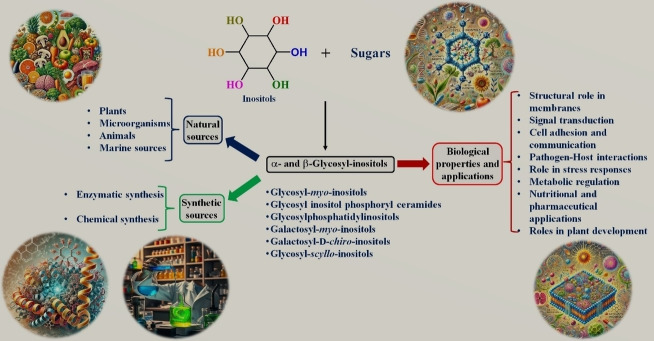

## 1 Introduction

The main aim of this review is to provide an updated summary of glycosyl inositols, with particular emphasis on (i) inositols, including their classification and stereochemistry; (ii) a brief overview of their occurrence in natural sources, biological activities, and applications; and (iii) methods for their synthesis, including both chemical and enzymatic approaches, illustrated with representative examples.

Glycosyl‐inositols are molecules composed of one or several α‐ or β‐D‐glycosyl residues and inositol or methyl‐inositol mainly.^[1]^ The specific sugar moiety and the position of glycosylation on the inositol ring can vary, leading to a wide range of glycosyl‐inositol derivatives with different chemical and biological properties.[^2^, ^3^, ^4^, ^5^, ^6^, ^7^, ^8^]

This review covers glycosyl‐*myo*‐inositols, including α‐ and β‐monoglycosyl‐*myo*‐inositols, α‐diglycosyl‐*myo*‐inositols, glycosyl inositol phosphoryl ceramides (GIPCs), glycosylphosphatidylinositols (GPIs), and phosphatidylinositol mannosides (PIMs). It also includes galactosyl‐inositols based on *myo*‐inositol (e. g., galactinol, digalactosyl‐*myo*‐inositol), and D‐*chiro*‐inositol (e. g., fagopyritol series A and B, methyl‐D‐*chiro*‐inositol, D‐ononitol). Additionally, examples of *scyllo*‐inositol glycosides are also covered.[^2^, ^3^, ^4^, ^5^, ^6^, ^7^, ^8^, ^9^, ^10^, ^11^, ^12^, ^13^, ^14^, ^15^, ^16^, ^17^, ^18^, ^19^, ^20^, ^21^, ^22^, ^23^, ^24^, ^25^, ^26^, ^27^, ^28^, ^29^, ^30^, ^31^, ^32^, ^33^, ^34^]

Most glycosylated forms of inositols are found in the plant kingdom; nevertheless, their low content, challenging extraction, separation, and detection have limited their isolation and commercial application.[^2^, ^3^, ^4^, ^5^, ^6^, ^7^, ^9^, ^10^, ^11^, ^12^, ^13^, ^14^, ^15^, ^16^, ^17^, ^18^, ^19^, ^20^, ^21^, ^22^, ^35^] To address the lack of a convenient source of these glycosides, chemical strategies[^1^, ^36^, ^37^, ^38^, ^39^, ^40^, ^41^, ^42^] and enzymatic tools[^43^, ^44^, ^45^, ^46^, ^47^, ^48^, ^49^, ^50^] for the synthesis of natural and unnatural glycosyl inositols have proven advantageous. Readers are encouraged to consult previous review articles and the primary literature cited herein to expand their understanding or interest in these compounds.[^51^, ^52^, ^53^, ^54^, ^55^, ^56^, ^57^, ^58^, ^59^, ^60^]

## 2 Inositols

### 2.1 Inositols: Classification, Structure, and Occurrence in Natural Sources

**Figure 1:**
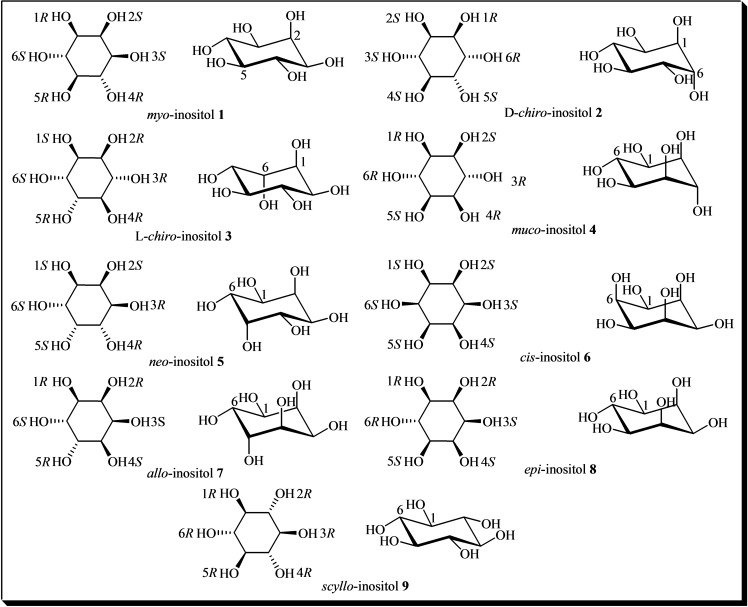
Structures of the nine stereoisomers of inositol.

Inositols (C₆H₁₂O₆) are cyclitols characterized by a cyclohexane ring where each carbon atom is bonded to a single hydroxyl group. This particular type of structure, with six stereogenic centers, implies the presence of 64 potential stereoisomers. However, due to symmetry, the actual number of isomers is nine, as illustrated in Figure 1. Five of these – *myo*‐**1**, D‐*chiro*‐**2**, *muco*‐**4**, *neo*‐**5**, and *scyllo*‐**9**‐inositol – occur naturally, while the other four isomers (L‐*chiro*‐**3**, *allo*‐**7**, *epi*‐**8**, and *cis*‐**6**‐inositol) are derived from *myo*‐inositol and obtained through chemical synthesis.[^61^, ^62^] In their most stable conformation, inositols adopt the chair conformation, positioning the maximum number of hydroxyl groups in the equatorial orientation to maximize their separation from each other.^[61]^ Inositols are widely present in plant‐based foods (fruits, vegetables, seeds, etc.), although they have also been detected in foods of animal origin at lower levels.[^63^, ^64^, ^65^, ^66^, ^67^, ^68^, ^69^, ^70^, ^71^, ^72^, ^73^, ^74^, ^75^, ^76^, ^77^, ^78^, ^79^, ^80^, ^81^, ^82^, ^83^, ^84^, ^85^, ^86^, ^87^, ^88^, ^89^] The most diffused form in nature is *myo*‐inositol **1**, followed by the optically active isomer D‐*chiro*‐inositol **2**.[^66^, ^72^, ^73^]

**Figure 2:**
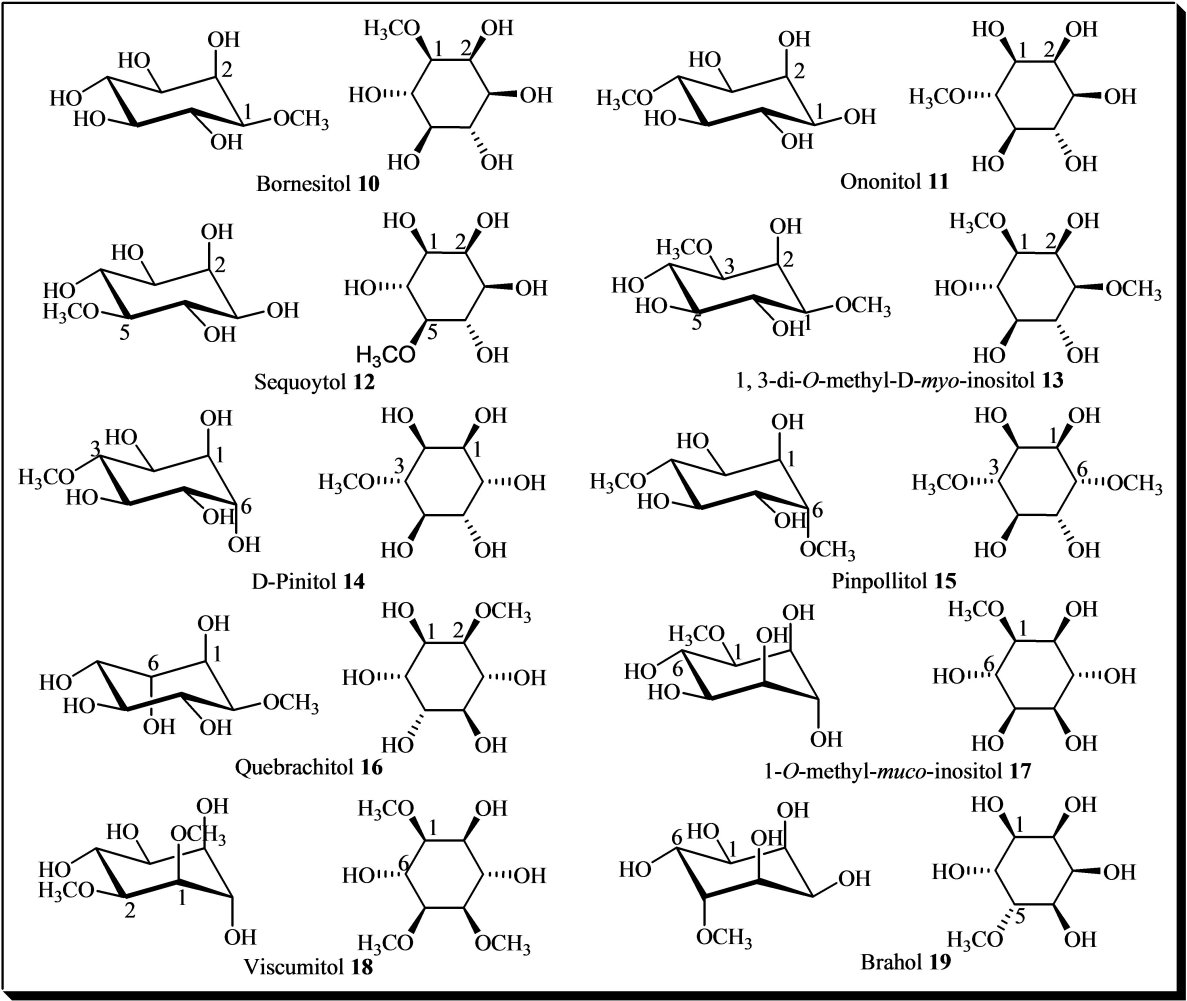
Methyl ethers derivatives of inositols. 1‐*O*‐methyl‐*myo*‐inositol **10**, 4‐*O*‐methyl‐*myo*‐inositol **11**, 5‐*O*‐methyl‐*myo*‐inositol **12**, 1,3‐di‐*O*‐methyl‐D‐*myo*‐inositol **13**, 3‐*O*‐methyl‐D‐*chiro*‐inositol **14**, di‐*O*‐methyl‐(+)‐*chiro*‐inositol **15**, 1 L‐2‐*O*‐methyl‐*chiro*‐inositol **16**, 1,2‐Di‐*O*‐methyl‐*muco*‐inositol **18**, 5‐*O*‐methyl‐*allo*‐inositol **19**.

Additional inositol derivatives include inositol methyl ethers (Figure 2), which are recognized as plant secondary metabolites.[^63^, ^64^, ^66^, ^67^, ^68^, ^69^, ^70^, ^71^] These compounds act as secondary metabolites in plants, helping them cope with environmental stresses, with D‐pinitol **14** being the most common inositol ether.^[66]^

### 2.2 Biological Properties of Inositols and Applications

Inositols play vital roles in various biochemical and metabolic functions across different tissues and organs.[^34^, ^35^, ^61^, ^62^, ^65^, ^66^, ^67^, ^73^, ^89^, ^90^] They are essential for cellular processes such as signal transduction (phosphatidylinositol 4,5‐bisphosphate),^[91]^ membrane formation (phosphatidylinositol and its phosphorylated derivatives),^[92]^ cell wall integrity (phosphatidylinositol),^[93]^ ion channel function (inositol 1,4,5‐trisphosphate),^[94]^ phosphate storage (inositol‐1,4,5‐trisphosphate),^[95]^ and osmoregulation (*myo*‐inositol).^[96]^ Each type of inositol plays a distinct role in these processes. Phosphatidylinositol 4,5‐bisphosphate acts as a second messenger in human signal transduction, mobilizing intracellular calcium crucial for processes such as secretion and cell proliferation.^[91]^ Phosphatidylinositol and its phosphorylated derivatives are key components of eukaryotic membranes, contributing to structure and fluidity.^[92]^ Phosphatidylinositol is vital for cell wall integrity in fungi, ^[93]^ while inositol hexakisphosphate (phytic acid) in plants serves as a phosphate storage molecule.^[95]^ *Myo*‐inositol functions as a compatible osmolyte, enhancing ion transport efficiency and antioxidant capacity in aquaculture species like tilapia under salinity stress.^[96]^ While many of these roles are endogenous and independent of dietary supplementation in humans, exogenous inositol supplementation is particularly beneficial in aquaculture, demonstrating its ability to improve stress tolerance and metabolic efficiency in fish. Understanding these roles provides valuable insights into both physiological processes and potential applications of inositol supplementation.[^91^, ^92^, ^93^, ^94^, ^95^, ^96^]

Inositols also have notable properties and are used as dietary supplements to support the management of conditions such as metabolic syndrome,^[97]^ polycystic ovarian syndrome,^[98]^ diabetes,^[99]^ obesity,^[100]^ hypertension,^[101]^ atherosclerosis,^[102]^ neurodegenerative disorders,^[103]^ and as antioxidants.^[104]^ While inositols are not classified as drugs, ongoing research explores their potential therapeutic applications. In this context, they possess anti‐atherogenic,^[66]^ anti‐inflammatory,^[6]^ anti‐cancer properties,^[105]^ and their potential against SARS‐CoV‐2 is being explored.^[106]^ Technologically, inositols enhance the thickening of agents in aqueous solutions,^[107]^ benefiting pharmaceuticals,^[108]^ cosmetics,^[109]^ and food formulations.^[110]^

## 3 Glycosyl‐Inositols

### 3.1 Glycosyl‐*Myo*‐Inositols

Among the natural derivatives of glycosyl cyclitols, glycosyl‐*myo*‐inositols are the most prevalent and characteristic.[^2^, ^3^, ^5^, ^6^, ^7^, ^13^, ^14^, ^15^, ^16^, ^35^, ^77^, ^111^] A notable member of this class is mycothiol **20**, a thiol derivative found in Actinomycetota. Mycothiol functions as a cysteine reservoir and plays a critical role in the detoxification of alkylating agents as well as reactive oxygen and nitrogen species.[^16^, ^35^] Another example of an α‐monoglycosyl‐*myo*‐inositol is mannosyl inositol **21**, which has been isolated from *Saccharomyces cerevisiae*.^[2]^

In addition, β‐glycosyl‐*myo*‐inositols constitute another significant category within this group. Notably, 6‐β‐galactinol **22** has been identified in the mammary glands of rats.^[5]^ Furthermore, two series of *myo*‐inositol‐derived glycolipid analogs, lanceolitols A1–A7 **23** and lanceolitols B1–B7 **24**, were isolated from the leaves of the Mexican medicinal plant *Solanum lanceolatum*. These lanceolitols demonstrated *in vivo* anti‐inflammatory activity.^[6]^

**Figure 3:**
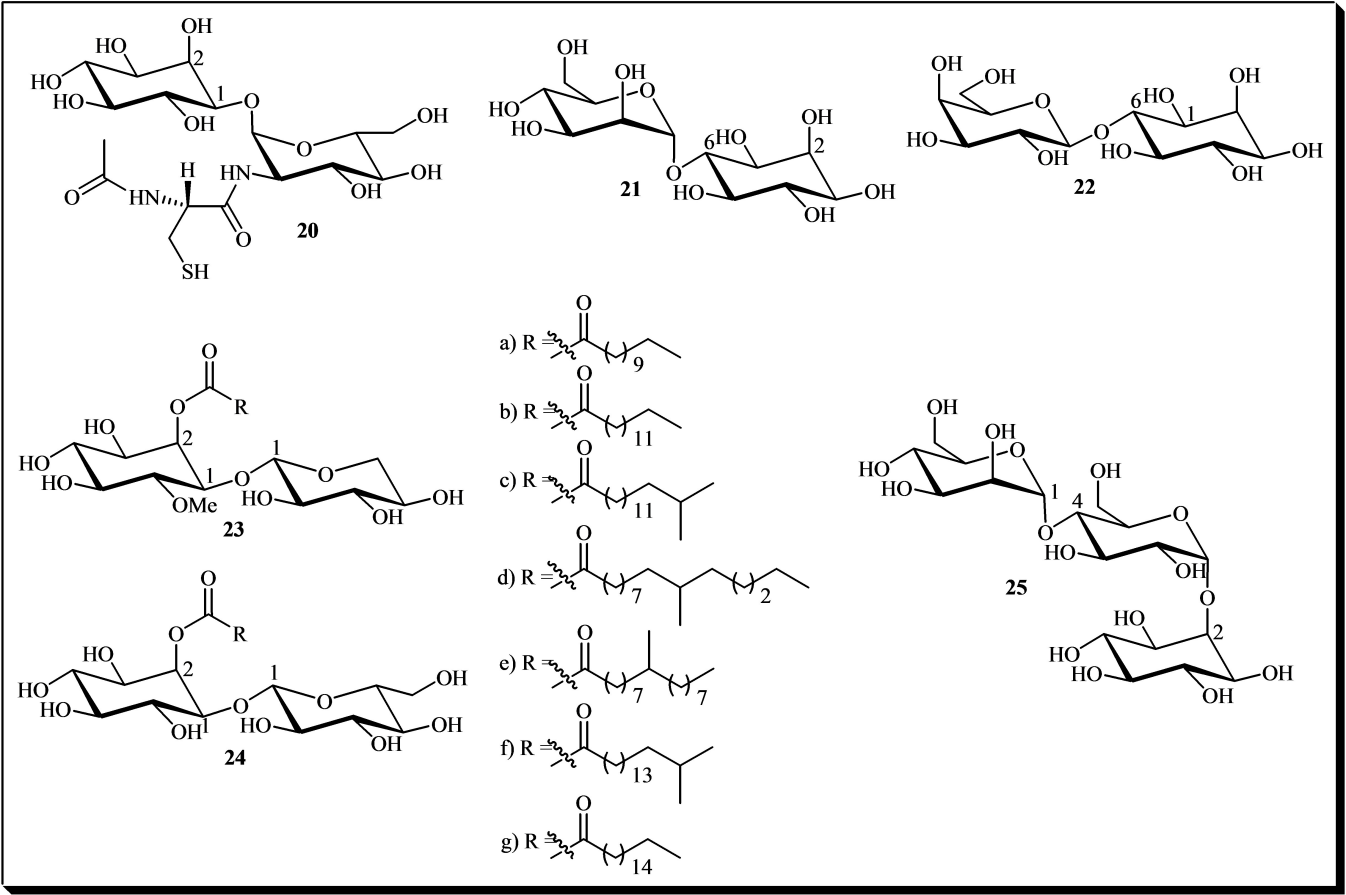
Chemical structures of glycosyl‐*myo*‐inositols **20**–**25**.

Additionally, Smith and Fry (1999) investigated the secretion of oligosaccharins from cultured Rosa cells and isolated an extracellular α‐diglycosyl‐*myo*‐inositol **25**. This derivative influences amino acid metabolism in plants.^[12]^ Figure 3 illustrates the chemical structures of glycosyl‐*myo*‐inositols **20**–**25** as described.

### 3.2 Glycosyl Inositol Phosphoryl Ceramides

**Figure 4:**
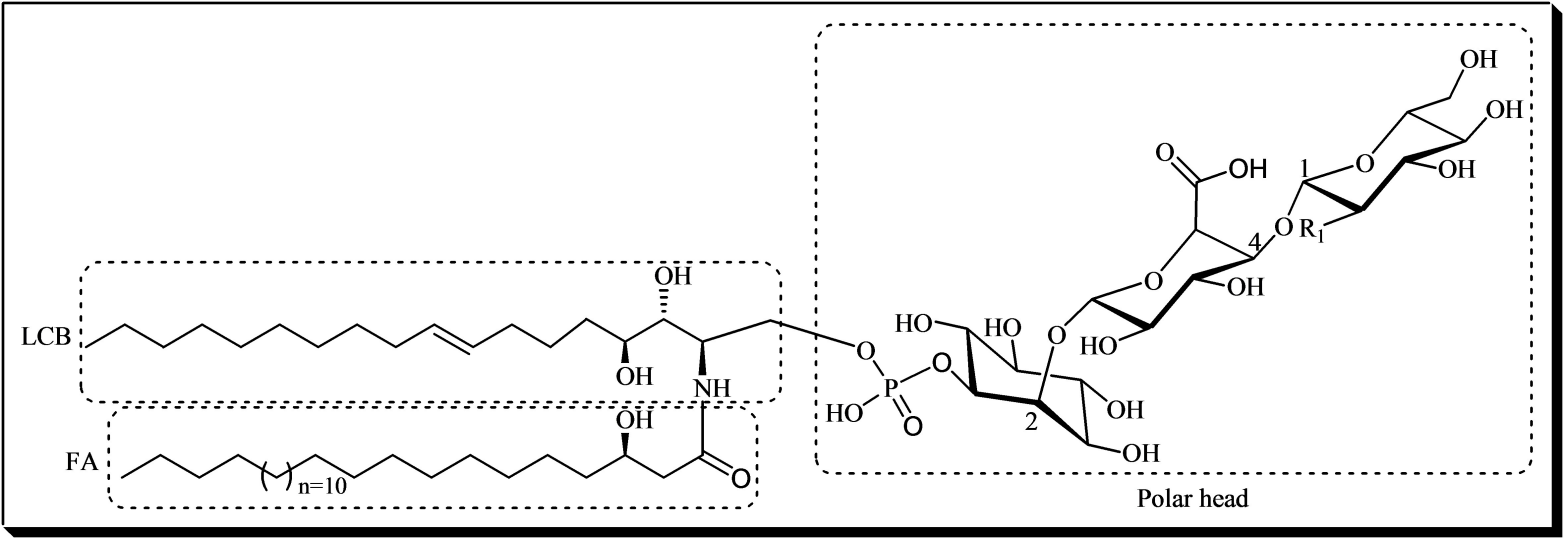
An example of a GIPC **26** structure, illustrating its three fundamental building blocks: fatty acid (FA), long‐chain base (LCB), and polar head. The R1 group in the polar head can vary, being either a hydroxyl group, an amine group, or an *N*‐acetylamine group.

Glycosyl inositol phosphoryl ceramides (GIPCs, Figure 4) **26** are major sphingolipids on Earth, accounting for a significant fraction of the total lipids in plants and fungi, which, in turn, represent a large portion of the Earth's biomass. The core structure of GIPCs comprises a ceramide moiety attached to an inositol–glucuronic acid unit through a phosphodiester bond. This foundational structure can be modified by the addition of various saccharides, resulting in compounds such as Galactose‐Glucose(R_1_)‐Glucuronic acid‐inositol‐1‐phosphoceramide in plants or Mannose‐inositol‐1‐phosphoceramide in fungi. GIPCs are crucial components in plant cellular physiology, where their diverse functions underscore their significance. These molecules contribute to membrane microdomain structuring and integrity, ensuring functionality under diverse environmental conditions. Their glycan residues play key roles in cell‐cell communication and pathogen recognition, linking structural stability with adaptive signaling mechanisms. Additionally, GIPCs are involved in responding to abiotic stresses and regulating programmed cell death associated with pathogen resistance. This dual role–structural and signaling–positions GIPCs as pivotal molecules in plants’ adaptive responses to environmental challenges, integrating cellular integrity with dynamic stress responses.[^14^, ^15^]

### 3.3 Glycosylphosphatidylinositols

**Figure 5:**
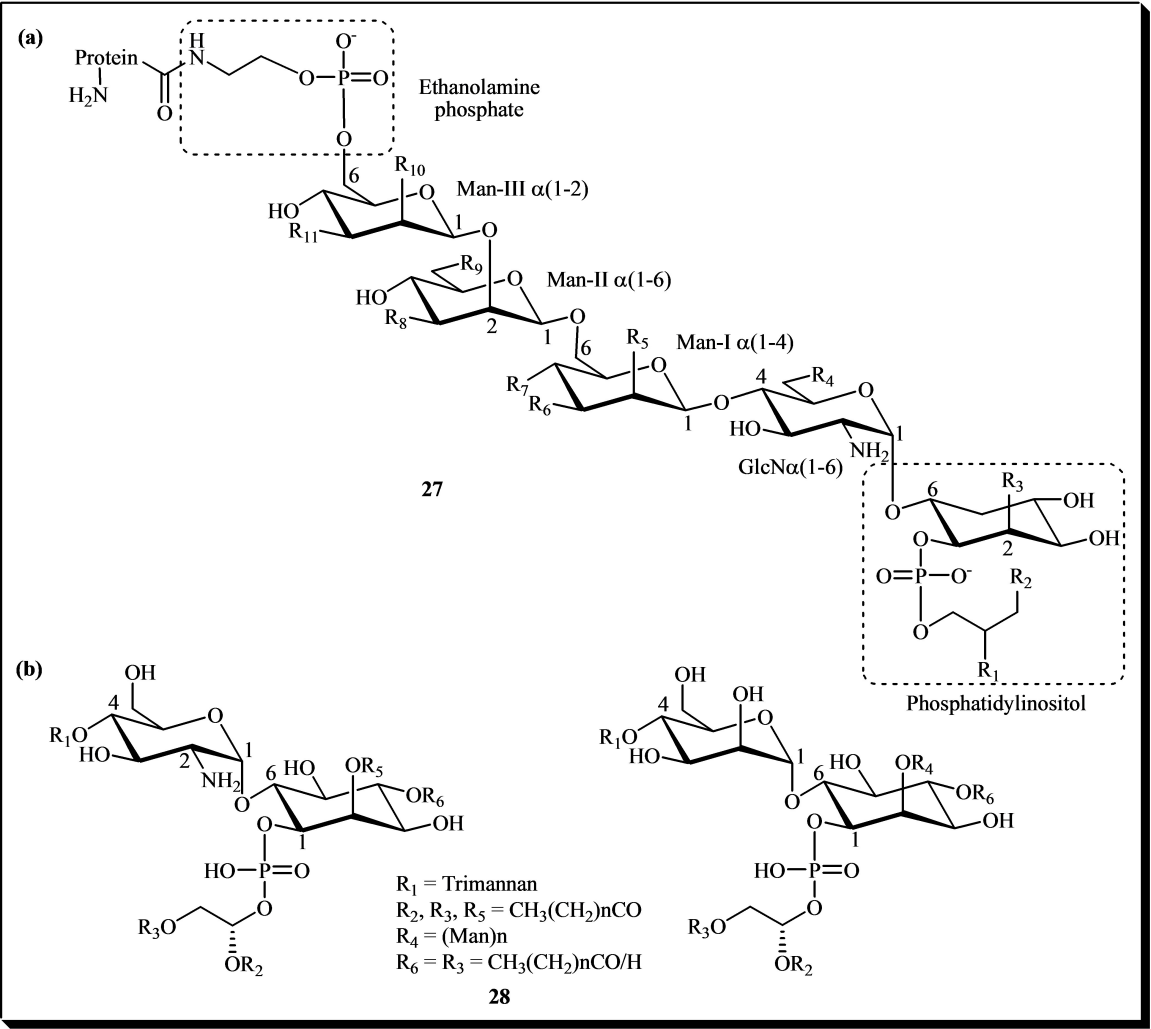
(a) GPI general structure and anchoring function. R_1_ and R_2_ may include long‐chain fatty acids, alkyl or alkenyl chains, or hydroxyl groups. R_3_, often a palmitate on C‐2 of inositol. R_4_ and R_9_ may carry ethanolamine phosphate groups, while R_5_–R_10_ can accommodate monosaccharides, oligosaccharides, or hydroxyl groups. In some anchors, ceramides replace glycerolipids, further diversifying their structure. (b) Chemical structures of phosphatidylinositol mannosides **28**.

Examples of more complex glycosylated *myo*‐inositols include glycosylphosphatidylinositols anchors (GPIs, **27**; Figure 5a)^[16]^ and phosphatidylinositol mannosides (PIMs, **28**; Figure 5b).^[21]^ The general structure of GPIs anchors features a conserved core: ethanolamine‐PO_4_–6Manα1–2Manα1–6Manα1–4GlcNα1–6*myo*‐inositol‐1‐PO_4_‐lipid. Structural variability arises from substitutions, represented by R groups (see Fig. 5a). These modifications allow GPIs to anchor proteins to the cell membrane, playing key roles in cellular signaling and membrane dynamics.^[112]^

GPIs have significant potential for pharmaceutical applications due to their chemical nature and unique biophysical and cell biological properties. They play a critical role in the survival and virulence of many parasites, making them a promising target for anti‐parasitic drug development.^[113]^ Numerous *in vitro* studies have demonstrated that these compounds exert partial insulin‐mimetic activity on glucose and lipid metabolism in insulin‐sensitive cells.[^17^, ^18^, ^20^] Other notable pharmaceutical uses include the production, extraction, and purification of therapeutic proteins, such as GPI‐anchored proteins for transport into target cells and antibodies;[^114^, ^115^] the development of novel biomaterials and biosensors for multi‐step catalysis;^[116]^ and applications in oral gene therapy.[^117^, ^118^, ^119^] Additionally, several GPIs are being investigated as potential malaria vaccine candidates.^[120]^

On the other hand, PIMs play a crucial role in host‐pathogen interactions during diseases such as tuberculosis and leprosy.^[23]^ Additionally, they are considered the structural foundation of lipoglycans, including lipomannan (LM) and lipoarabinomannan (LAM), due to their function as essential precursors in their biosynthesis. PIMs are glycolipids composed of a phosphatidyl‐*myo*‐inositol anchor attached to one to six mannose residues. These molecules can also have varying degrees of acylation. The structure of PIMs forms the backbone for the sequential addition of mannose and arabinose residues during the biosynthesis of LM and LAM. PIMs are extended by the addition of a mannose‐rich α‐(1→6)‐linked backbone. This backbone is further modified with branching α‐(1→2)‐mannose residues to produce LM. LM undergoes additional modifications, where arabinan chains are appended to form LAM. The arabinan component includes a highly branched structure that defines LAM's biological functions.[^121^, ^122^]

The synthesis of LM and LAM begins with the mannosylation of PIMs at the plasma membrane, mediated by mannosyltransferases. These enzymes catalyze the stepwise addition of mannose and other sugar residues. Thus, PIMs provide the foundational scaffold on which the more complex structures of LM and LAM are built, underscoring their significance in the biosynthesis and function of these key mycobacterial lipoglycans.[^121^, ^122^]

### 3.4 Galactosyl‐*Myo*‐Inositols

Seeds from numerous species store mono‐galactosyl‐cyclitols as part of their natural maturation compounds. Among these, galactinol **29** is extensively researched and well‐recognized. Initially isolated from sugar beet juice, galactinol **29** has been identified in various plant organs across a wide range of species.^[3]^ This compound plays a crucial role as a galactosyl donor in the biosynthesis of glycosinolates.[^3^, ^9^] Other cyclitol mono‐galactosides include galactosyl‐bornesitol (also known as lathyritol) **30**, which was isolated from sweet pea seeds,^[4]^ and ononitol **31**, identified in adzuki beans.^[10]^

Cyclitol digalactosides have also been discovered in natural sources. These include digalactosyl‐ononitol **32**, found in adzuki beans,^[10]^ α‐digalactosyl‐sequoyitol **33**, present in alfalfa,^[3]^ and α‐digalactosyl‐L‐*myo*‐inositol **34**, isolated from common buckwheat (*Fagopyrum esculentum Moench*) seeds.^[13]^

**Figure 6:**
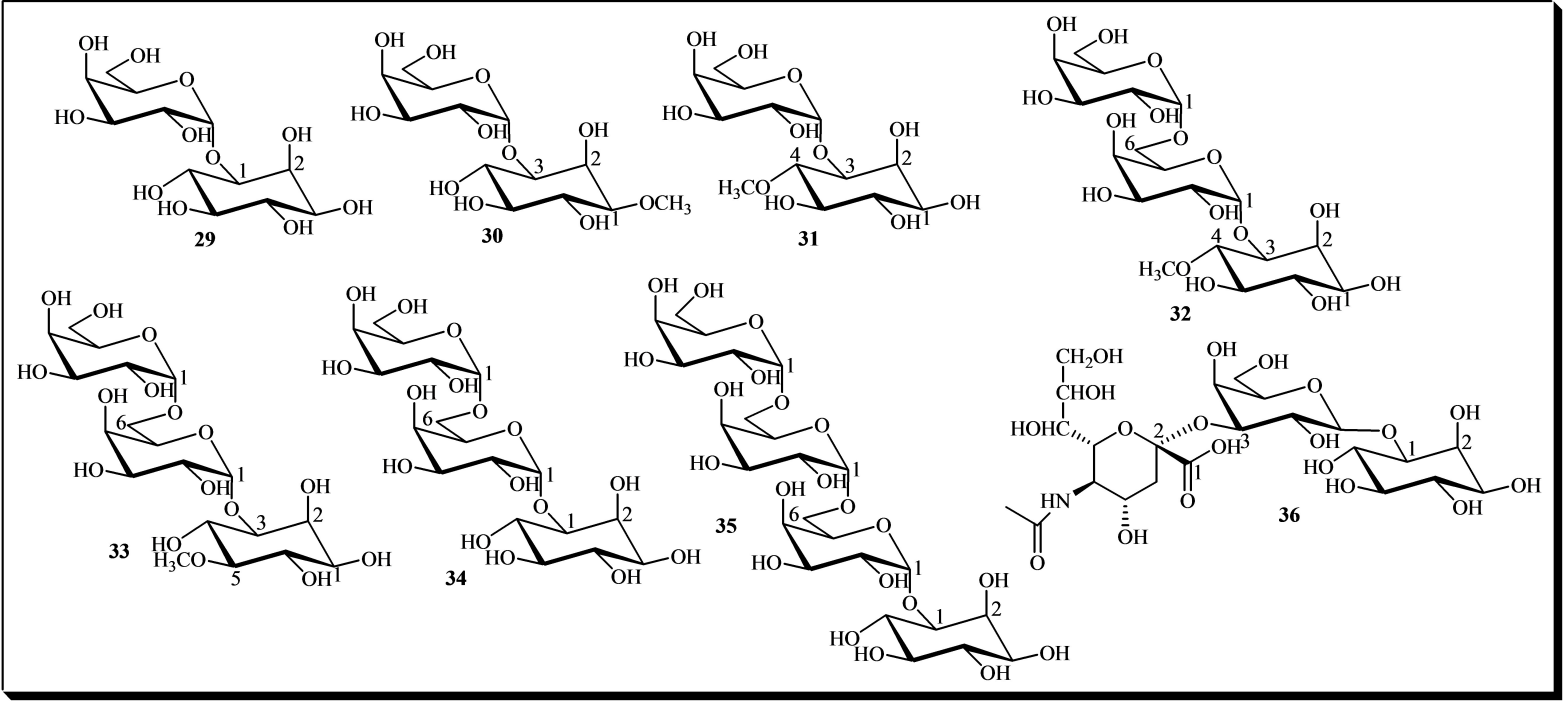
Chemical structures of galactosyl‐*myo*‐inositols **29**–**36**.

Another class of biomolecules containing an inositol moiety is represented by trigalactosyl‐*myo*‐inositol **35**, which was isolated from common buckwheat seeds.[^13^, ^29^] In contrast, a *myo*‐inositol glycoside known as neuraminyl‐galactinol derivative **36** has been identified in human urine.^[7]^ Figure 6 illustrates the structures of the galactosyl‐*myo*‐inositols **29**–**36** mentioned above.

### 3.5 Galactosyl‐D‐*Chiro*‐Inositols

**Figure 7:**
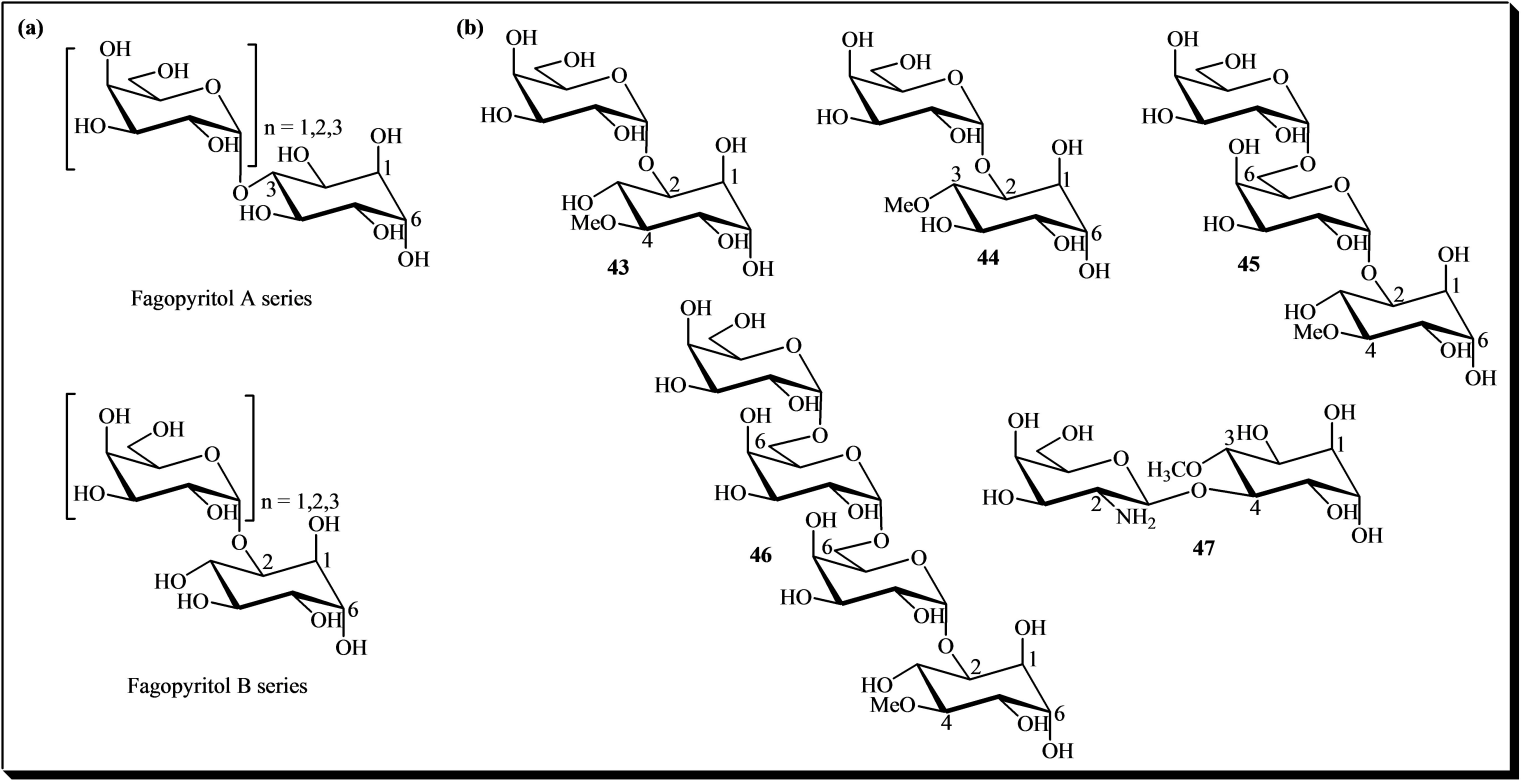
(a) Fagopyritols mono‐, di‐ and tri‐α‐D‐galactopyranosyl‐D‐*chiro*‐inositols (compounds **37**–**42**). n=1, fagopyritols A1 and B1; n=2, fagopyritols A2 and B2; n=3, fagopyritols A3 and B3. (b) Methyl‐D‐*chiro*‐inositol‐galactose conjugate.

Galactosyl‐D‐*chiro*‐inositols represent a specific class of glycosylated inositol derivatives found in nature, with implications for both plant biology and potential applications in human health and nutrition research.[^13^, ^24^, ^25^, ^26^] This group includes the fagopyritols (see Figure 7a), which are categorized into two series (A and B) based on the linkage between galactopyranosyl and D‐*chiro*‐inositol. In the fagopyritol A series, the α‐galactoside linkage is to the 3‐carbon of D‐*chiro*‐inositol, while in the fagopyritol B series, it is to the 2‐carbon of the cyclitol. The series include fagopyritol A1 and fagopyritol B1 (mono‐galactosyl D‐*chiro*‐inositol isomers **37** and **38**), fagopyritol A2 and fagopyritol B2 (di‐galactosyl D‐*chiro*‐inositol isomers **39** and **40**), and fagopyritol B3 (tri‐galactosyl D‐*chiro*‐inositol isomers **41** and **42**). The galactosyl residues in the di‐ and tri‐galactosyl D‐*chiro*‐inositol isomers are connected by α(1→6) linkages. Fagopyritols and/or D‐*chiro*‐inositol mixtures have demonstrated notable activities, including anti‐diabetic effects,^[27]^ antioxidant properties,^[123]^ and anti‐inflammatory benefits.^[124]^ Additionally, fagopyritols are associated with enhanced tolerance to desiccation and improved storability of buckwheat seeds.^[77]^

On the other hand, the analysis of soluble carbohydrates in plants has uncovered α‐galactosides derived from methyl‐inositol derivatives. Among these compounds, methyl‐D‐*chiro*‐inositol monogalactosyl conjugates, such as galactopinitol A **43** and galactopinitol B **44**, have been identified in soybeans.[^28^, ^29^, ^30^, ^125^] Additionally, digalactosyl‐ciceritol **45** has been found in chickpeas (*C. arietinum*), lentils (*Lens esculenta*), and lupins (*Lupinus albus*).^[31]^ In contrast, trigalactosyl‐methyl‐D‐*chiro*‐inositol **46** has been detected in lentils, alfalfa, and soybeans.^[32]^

Another category of biomolecules featuring the D‐*chiro*‐inositol moiety is exemplified by galactosamine‐D‐*chiro*‐inositol **47**, isolated from beef liver. It acts as a pseudo‐disaccharide Mn^2+^ chelate with insulin‐like activity.^[33]^ Figure 7 illustrates the chemical structures of compounds **36**–**47**.

### 3.6 Glycosyl‐*Scyllo*‐Inositols

**Figure 8:**
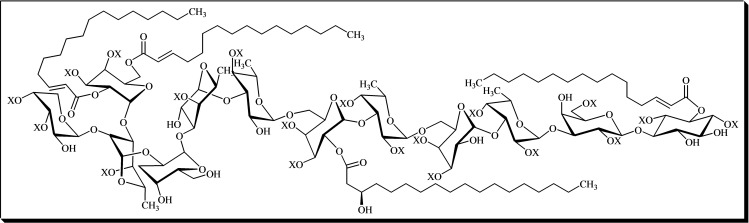
Structure of axinelloside A, X=SO_3_Na.

*Scyllo*‐inositol is a rare polyol found in natural products, notably present in human brains. Glycosyl‐*syllo*‐inositols, such as those in Axinelloside A **48** (Figure 8), are complex glycolipids that play key roles in cellular biology. Axinelloside A, a sulfated saccharide isolated from the lipophilic extract of the Japanese marine sponge *Axinella infundibula*, has been reported to exhibit potent inhibitory effects against human telomerase *in vitro*. Inhibiting telomerase activity shows promise as a therapeutic approach for cancer treatment, as over 85 % of human cancers express telomerase, while this enzyme is generally absent in most somatic cells.^[8]^ However, the structural and mechanistic details of how Axinelloside A interacts with telomerase remain unclear.[^126^, ^127^]

Another interesting *scyllo*‐inositol‐containing saccharide has been identified in human urine. This glycoside features an *O*‐α‐*N*‐acetylneuraminosyl‐(2→3)‐*O*‐β‐D‐galactopyranosyl residue attached to *scyllo*‐inositol **9**, although the specific glycosylation position on the cyclitol was not disclosed by the authors.^[128]^

## 4 Chemical Synthesis of Glycosyl Inositols

The diverse and complex structures of glycosyl inositols, combined with their important biological properties, present a significant challenge for synthetic chemists. The chemical synthesis of glycosyl inositols typically involves several complexities: the regiospecific protection of inositol and glycosyl residues, optical resolution of resulting intermediates, precise control of regioselectivity and stereoselectivity during glycosylation of the inositol backbone to obtain specific isomers, complete deprotection of all protecting groups, and subsequent purification of the glycosylated compounds.[^129^, ^130^, ^131^] Nonetheless, numerous effective glycosylation strategies have been developed to address these challenges, continually advancing in refinement and innovation.[^111^, ^132^, ^133^, ^134^, ^135^, ^136^, ^137^, ^138^, ^139^]

In general, the chemical synthesis of glycosides involves monomeric sugar units (glycosyl donors) linked via *O*‐glycosidic bonds to a glycosyl acceptor. This process includes: (a) activation of a glycosyl donor equipped with a leaving group at the anomeric carbon, and (b) efficient, selective coupling with the glycosyl acceptor, promoted by an activator.^[140]^ The glycosidic bond forms through the nucleophilic displacement of the leaving group at the anomeric carbon by an alkoxy group of an R‐OH alcohol or a partially protected sugar.^[141]^

The regioselective protection of hydroxyl groups in inositols is crucial for the chemical synthesis of glycosyl inositols and other derivatives. This step controls reactivity and stereochemistry in subsequent reactions, such as glycosylation or modifications. The six hydroxyl groups of inositol pose a challenge for achieving selectivity due to their similar chemical properties and spatial arrangement.^[142]^

### 4.1 Precursors for the Chemical Synthesis of Glycosyl Inositols: Protected Inositols

The first key intermediates for synthesizing biologically important glycosyl inositols are hydroxyl group‐protected derivatives, which retain free hydroxyl group(s) at specific positions.[^129^, ^143^] Protected inositols have been derived from naturally occurring cyclitols and their derivatives.[^130^, ^144^, ^145^]

The regioselective protection of inositols and the selective functionalization of their six hydroxyl groups are primarily influenced by the carbocyclic ring structure, intramolecular and intermolecular interactions (such as hydrogen bonding and metal ion chelation), the acidity of the free hydroxyl groups, neighboring functional groups, the nature of the protecting groups, and the choice of solvents in the reaction mixture. Chemical modifications at specific positions on the inositol ring can impact the reactivity of other hydroxyl groups by altering charge distribution, hydrogen bonding patterns, conformational dynamics, chelation interactions, or steric hindrance, thereby influencing their relative reactivities.[^145^, ^146^, ^147^, ^148^]

Syntheses starting from meso‐inositol typically require chemical or enzymatic resolution to obtain enantiomerically pure products. Several methods exist for desymmetrizing symmetric inositol derivatives, with most studies focusing on *myo*‐inositol due to its low cost and availability.[^149^, ^150^, ^151^, ^152^]

In general, hydroxyl groups in inositols are protected as ketals,^[153]^ benzylidene derivatives,^[59]^ direct allylation,^[154]^ or as isopropylidene derivatives.^[155]^ In *myo*‐inositol, the C1‐, C3‐, and C5‐hydroxyl groups are often protected as ortho esters,^[156]^ while in *scyllo*‐inositol similar protection strategies are employed.^[157]^ Experimental results indicate that, in *myo*‐inositol, the reactivity order of hydroxyl groups is C1~C3>C4>C5. For *myo*‐ and *chiro*‐inositol, equatorial hydroxyl groups adjacent to axial hydroxyl groups are most reactive toward acylation and alkylation.[^158^, ^159^]

The challenges of resolving racemic derivatives and achieving regioselectivity in hydroxyl group protection are less pronounced in D‐ and L‐*chiro*‐inositols, owing in part to their C2 symmetry and the presence of two less‐reactive axial hydroxyl groups. In contrast, for *scyllo*‐inositol, selective monoprotection is straightforward since all hydroxyl groups are equivalent.[^160^, ^161^]

The distinct structural features of inositols play a critical role in determining the strategies employed for their chemical modification and functionalization. These differences, including the regioselectivity challenges in protecting hydroxyl groups and the inherent symmetry of specific inositol isomers, directly influence the design of synthetic pathways. Building on this foundation, the next section explores the chemical synthesis routes for glycosyl inositols, highlighting the significance of protected inositols as key intermediates in the preparation of both natural and synthetic derivatives. For more detailed information on these approaches, readers are encouraged to consult the references cited in this review.[^1^, ^37^]

### 4.2 A Chemical Method for Synthesizing Fagopyritols A1 and B1

**Figure 9:**
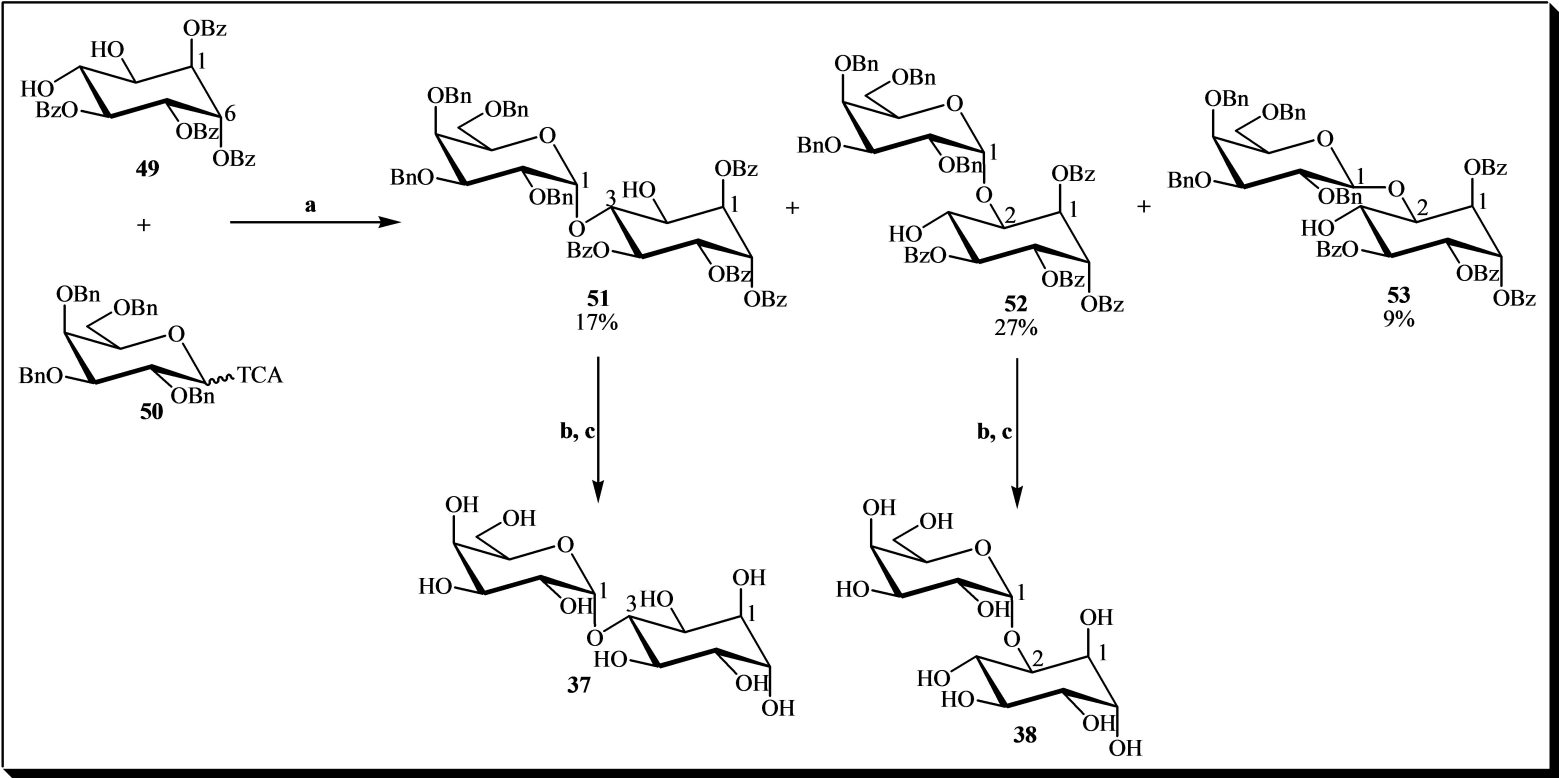
Synthesis of fagopyritols A1 and B1. Reagents and conditions: (a) ethyl ether, TMSOTf, −40 to −5 °C, 1 h; (b) MeONa/MeOH 1 M, MeOH/THF, rt, 10 min, quantitative; (c) H_2_ Pd/C, EtOH/MeOH/H_2_O, rt, overnight, quantitative. Percentages in the chemical structures represent the yield of each product formed.

Naturally occurring galactosylcyclitols play a vital role in plant physiology, especially in seed desiccation tolerance, as previously mentioned. Many of these natural products contain the optically active inositol isomer D‐*chiro*‐inositol **2**.^[77]^ Examples include fagopyritols A1 and B1, which have been synthesized with high yield through glycosylation of the diequatorial diol 1,4,5,6‐tetra‐*O*‐benzoyl‐D‐*chiro*‐inositol **49** using 2,3,4,6‐tetra‐*O*‐benzyl‐D‐galactopyranosyl trichloroacetimidate **50** as the glycosyl donor, followed by standard debenzoylation and catalytic hydrogenolysis (see Figure 9). During this process, D‐*chiro*‐inositol β‐galactoside **53** was produced as a byproduct. However, conducting the reaction at −78 °C resulted in pseudodisaccharide **52** being obtained in a 66 % yield as the sole glycosylation product.^[1]^

### 4.3 Appel–Lee Synthesis of α‐D‐Glycosyl‐*Myo*‐Inositols

**Figure 10:**
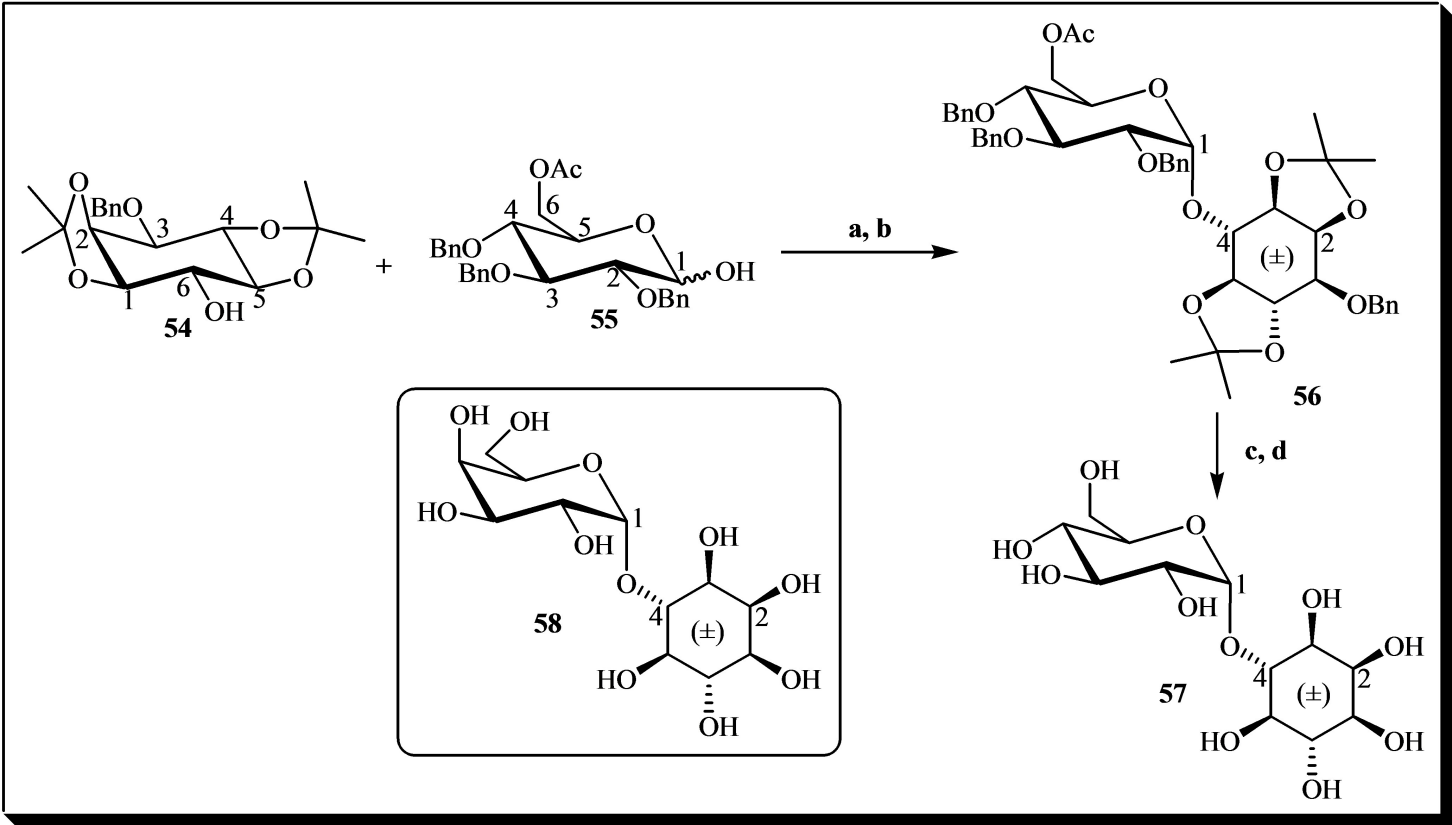
Synthesis of α‐D‐glucopyranosyl‐(1→4)‐(DL)‐*myo*‐inositol **57**. Reagents and conditions: (a) PPh_3_, CBr_4_, CH_2_Cl_2_, rt, 3 h; (b) tetramethylurea, (±)‐3‐*O*‐benzyl‐1,2 : 4,5‐di‐*O*‐isopropylidene‐*myo*‐inositol, rt, 1 week; (c) 1 atm H_2_, 10 % Pd/C, CH_3_OH, rt, 2 days; (d) CH_3_ONa/CH_3_OH, rt, 2 h.

Another notable instance of chemically synthesized monoglycosyl inositols is documented by Daniellou and Palmer, 2006. They successfully prepared α‐D‐glucopyranosyl‐(1→4)‐(DL)‐*myo*‐inositol **57** and α‐D‐galactopyranosyl‐(1→4)‐(DL)‐*myo*‐inositol **58** using the Appel‐Lee protocol. The synthetic pathway for **57**, depicted in Figure 10, utilized protected inositol **54** as the glycosyl acceptor and precursor **55** as the glycosyl donor. Subsequent steps involving hydrogenolysis, deprotection of benzyl esters, and methanolysis of the ester yielded glycoside **57** in quantitative yield.^[37]^

### 4.4 Synthesis of D‐Galactosaminopyranosyl‐D‐*Chiro*‐Inositols

**Figure 11:**
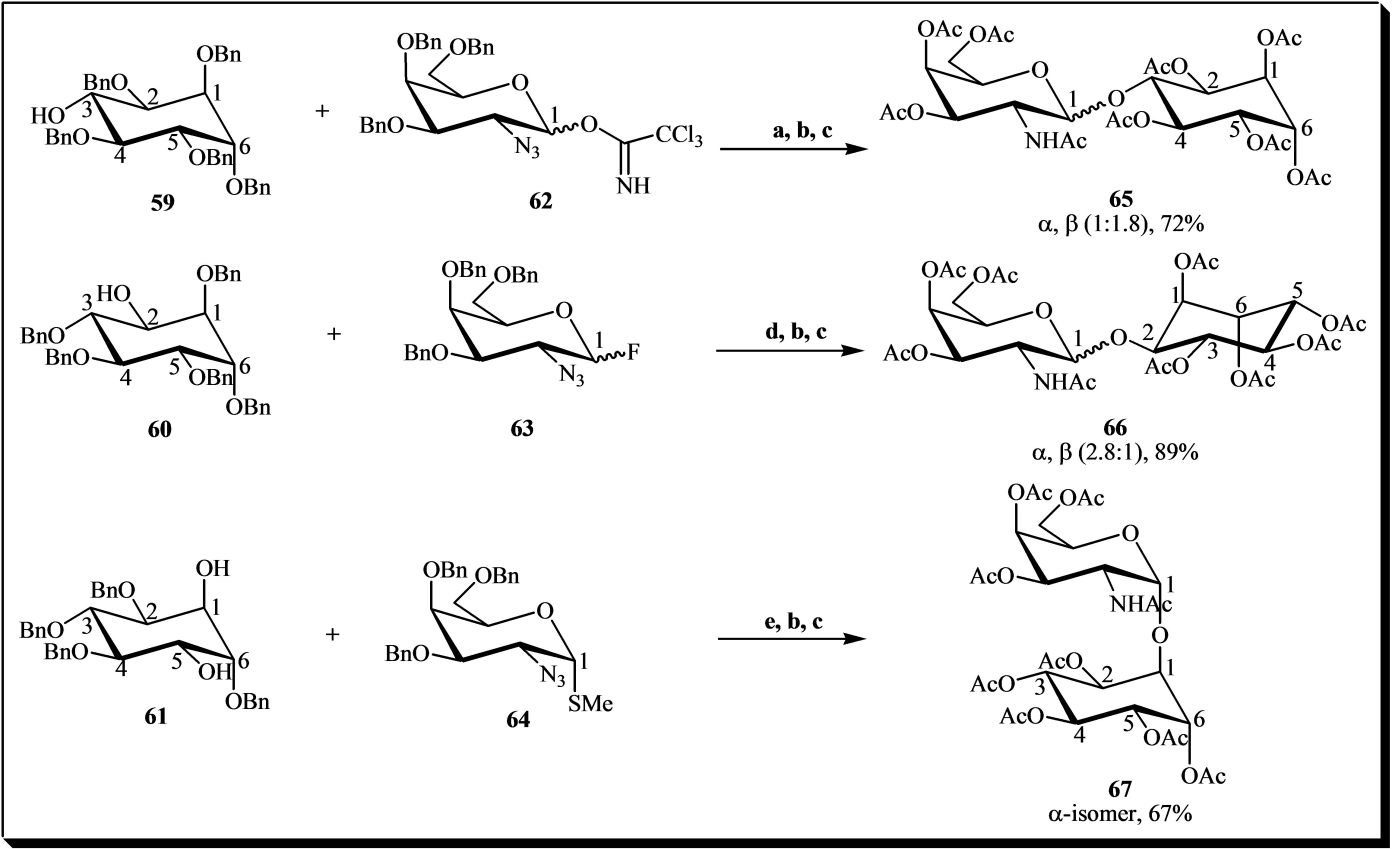
General scheme for the synthesis of D‐galactosaminopyranosyl‐D‐*chiro*‐inositols. Reagents and conditions: (a) TMSOTf, 4 Å MS, Et_2_O, −78 °C (72 % yield); (b) Na, NH_3_ (*l*), −78 °C; (c) Ac_2_O, Et_3_N, DMAP, THF, DMF; (d) AgOTf, Cp_2_ZrCl_2_, toluene, 4 Å MS, −42 °C to rt (89 % yield); (e) AgOTf, PhSeCl, 4 Å MS, toluene, −42 °C. The percentages in the chemical structures represent the yield of each product formed.

Isomeric D‐galactosaminopyranosyl‐D‐*chiro*‐inositols are compounds structurally akin to inositol phosphoglycans, a class of oligosaccharides believed to play a role in insulin signal transduction. These compounds have been synthesized (Figure 11) by glycosylating appropriate penta‐*O*‐benzyl‐D‐*chiro*‐inositols (glycosyl acceptors **59**, **60**, and **61**) with various glycosyl donors (**62**, **63**, and **64**). The condensation reaction yielded the isomers 2‐acetamido‐2‐deoxy‐D‐galactopyranosyl‐D‐*chiro*‐inositols **65**, **66**, and **67**, in yields ranging from 67 % to 89 %.^[38]^

### 4.5 Synthesis of the *Trypanosoma Cruzi* Lipopeptidophosphoglycan Heptasaccharyl *Myo*‐Inositol

**Figure 12:**
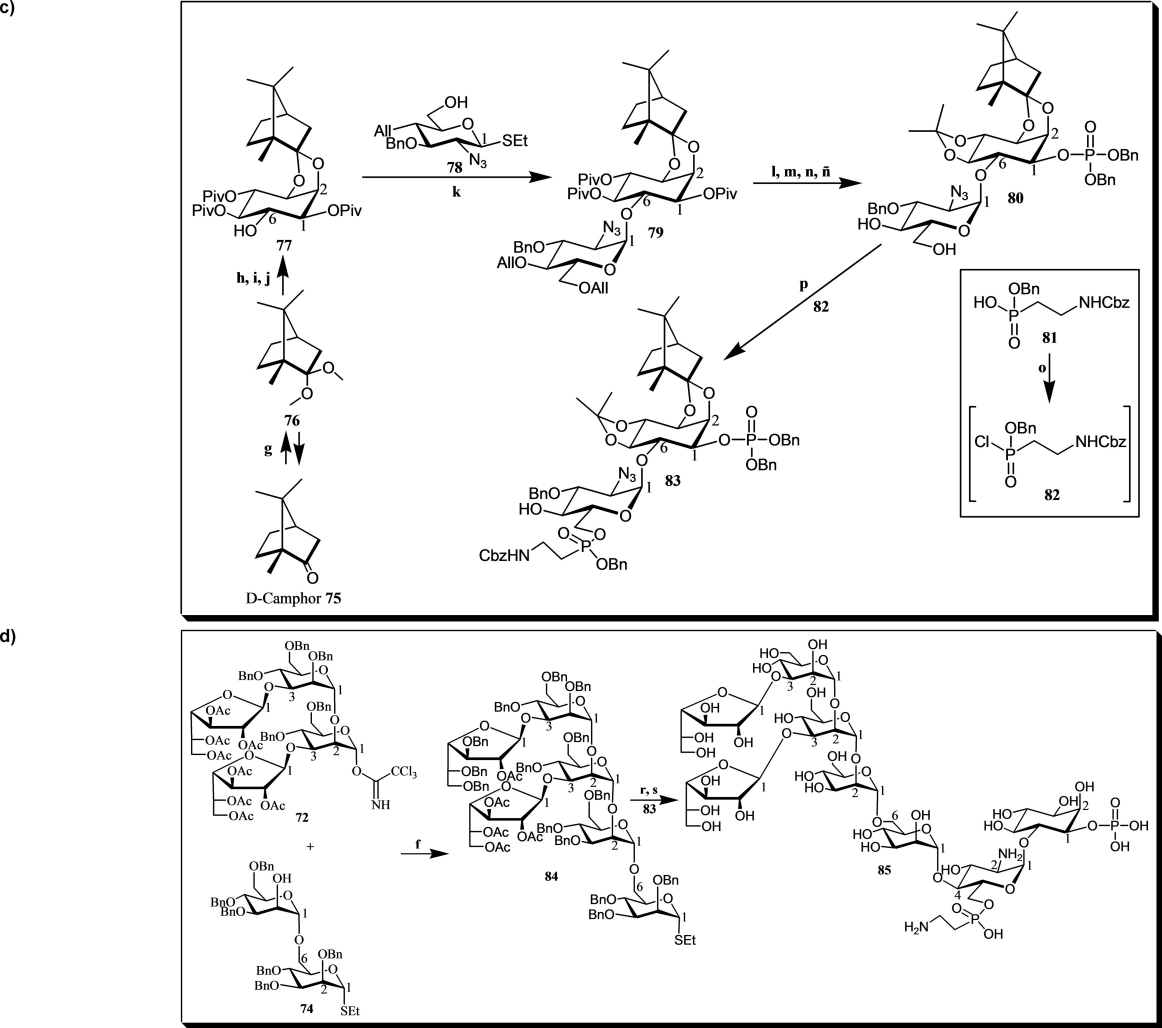
a) Building block **72** for the synthesis of the *T. cruzi* LPPG heptasaccharyl *myo*‐inositol **85**. Reagents and conditions: (a) SnCl_4_, 4 Å molecular sieves, −50 °C → rt, CH_2_Cl_2_; (b) 1. NaOMe, MeOH/CH_2_Cl_2_ (1 : 2); 2. BnBr, KI, Ag_2_O, DMF; (c) Br_2_, CH_2_Cl_2_; (d) AgOTf, 4 Å molecular sieves, CH_2_Cl_2_, −30 °C; (d) 1. *n*‐Bu_4_NIO_4_, TfOH, H_2_O, acetonitrile; 2. Cl_3_CCN, DBU, CH_2_Cl_2_. b) Building block **74** for the synthesis of the *T. cruzi* LPPG heptasaccharyl *myo*‐inositol **85**. Reagents and conditions: (e) NaOMe, CH_2_Cl_2_/MeOH (2 : 1). c) Building block **83** for the synthesis of the *T. cruzi* LPPG heptasaccharyl *myo*‐inositol **85**. Reagents and conditions: (g) MeOH, trimethyl orthoformate H_2_SO_4_; (h) *myo*‐inositol, H_2_SO_4_, DMSO; (i) CHCl_3_/MeOH/H_2_O (50 : 16 : 1), *p*‐TsOH; (j) ref. [28]; (k) MeOTf, Et_2_O, 4 Å molecular sieves; (l) NaOH, MeOH, reflux; (m) 2,2‐dimethoxypropane, pyridinium *p*‐toluenesulfonate, DMF; (n) (1) dibenzyl diisopropyl phosphoramidite, 1H‐tetrazole, CH_2_Cl_2_, (2) *m*‐CPBA, 0 °C; (ñ) (1) H_2_ (*g*), [bis(methyldiphenylphosphine)](1,5‐cyclooctadiene) Ir(I)PF_6_, THF, (2) NIS, H_2_O; (o) COCl_2_, cat. DMF, CH_2_Cl_2_; (p) TEA, CH_2_Cl_2_, 0 °C. d) A general framework employing a convergent building block strategy for the synthesis of the *T. cruzi* LPPG heptasaccharyl *myo*‐inositol **85**. Reagents and conditions: (f) TMSOTf, Et_2_O, 4 Å molecular sieves; (r) DMTST, 4 Å molecular sieves, Et_2_O; (s) 1. NaOMe, CH_2_Cl_2_/MeOH (2 : 1); 2. Na (*s*), NH_3_ (*l*); 3. 0.1 *M* HCl.

A notable chemical method was developed for the synthesis of heptasaccharyl *myo*‐inositol **85** (Figures 12a–d), a component of the lipopeptidophosphoglycan (LPPG) found in *Trypanosoma cruzi*. This compound plays a crucial role in the pathogenesis of Chagas disease and is of interest for both understanding parasite‐host interactions and developing therapeutic strategies. The synthesis strategy primarily involved the convergent assembly of three key building blocks: derivatives **72**, **74**, and **85**.[^39^, ^162^, ^163^]

The synthesis of compound **72** (see Figure 12a) began with the regioselective glycosylation of diol **68** using penta‐*O*‐acetyl‐β‐D‐galactofuranose **69**, where the 2‐*O*‐acetyl group of the galactofuranoside directed the reaction to form a 1,2‐*trans* glycosidic linkage. The acetates in **70** were then replaced with benzyl groups, resulting in the fully benzylated compound **71**. Subsequently, **71** was converted into a bromoglycoside through treatment with bromine, followed by glycosylation using silver trifluoromethanesulfonate to obtain the desired tetrasaccharide α‐trichloroacetimidate **72**. In a subsequent step, block **74** (see Figure 12b) was synthesized by reacting mannopyranoside **73** with sodium methoxide in a mixture of CH_2_Cl_2_‐CH_3_OH (2 : 1).^[39]^

In contrast, the synthesis of building block **83** (see Figure 12c) began with D‐camphor **75**, which was converted to dimethyl D‐camphor acetal **76**. This intermediate was then reacted with D‐*myo*‐inositol to yield the partially protected *myo*‐inositol derivative **77**. This approach provided a chiral D‐camphor *myo*‐inositol acceptor with a remaining C‐6 hydroxyl group available for further elongation using a suitable glucosamine donor. Glycosylation of derivative **77** with compound **78** produced the pseudo‐disaccharide **79**.[^39^, ^162^, ^163^] Subsequently, protecting group manipulations were carried out by cleaving the pivaloylic groups and introducing the isopropylidene acetal, resulting in a derivative with the inositol‐1‐OH phosphorylation site. Phosphorylation and deallylation were then accomplished to yield compound **80**. Finally, derivative **83** was synthesized by reacting compound **80** with the reactive phosphonochloridate **82**, which was derived from compound **81**.[^39^, ^162^]

In the next stage, the hexasaccharide donor **84** (see Figure 12d) was formed by condensing **72** and **74**, followed by glycosylation with derivative **83** to yield a fully protected heptasaccharide. The subsequent steps included deprotection, deacetylation, and debenzylation to obtain heptasaccharyl *myo*‐inositol **85**.[^39^, ^162^, ^163^]

### 4.6 Chemoenzymatic Synthesis of Fagopyritol Β‐Analogues

**Figure 13:**
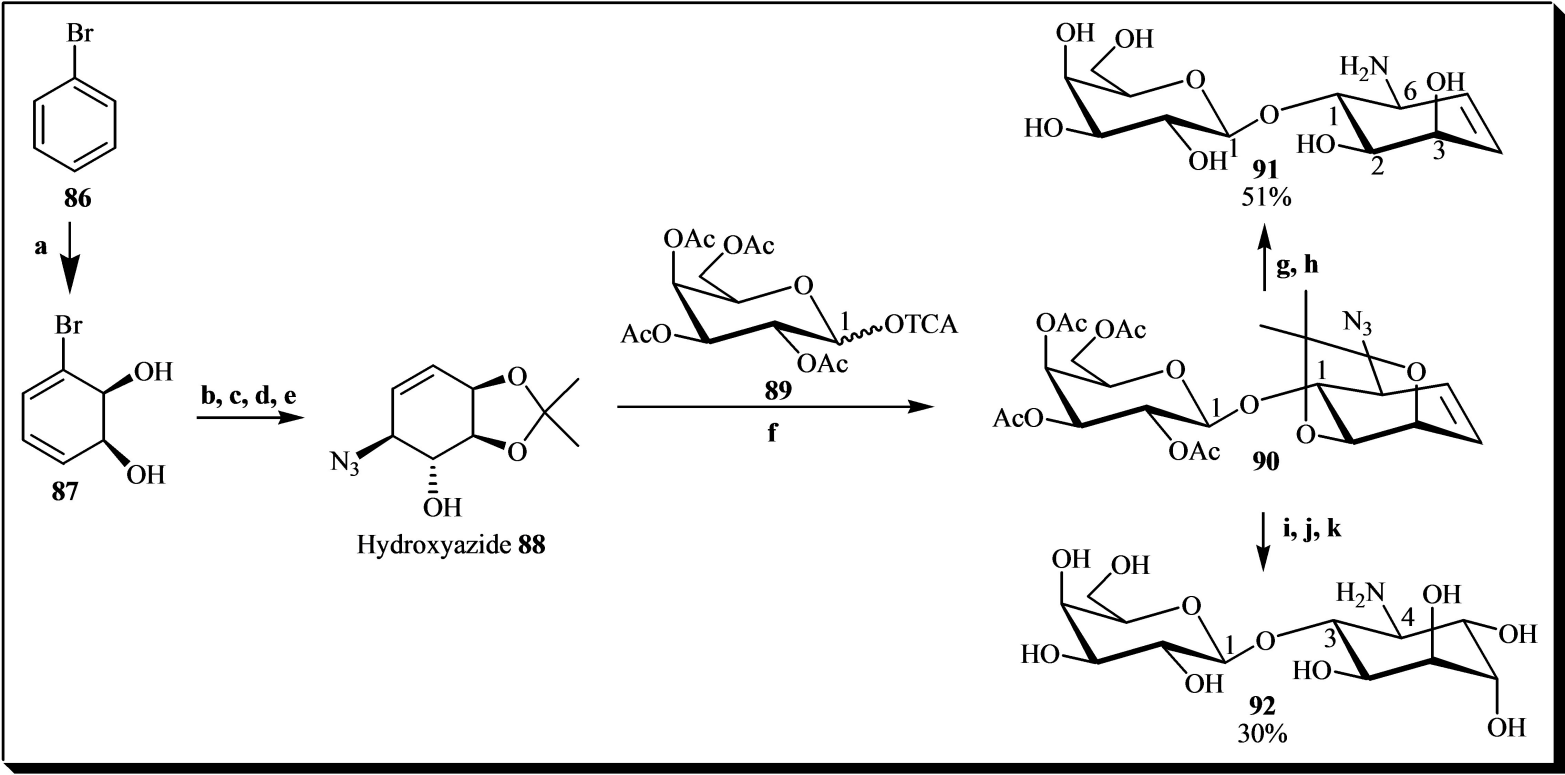
General sequence for the synthesis of fagopyritol β‐analogues. Reagents and conditions: (a) *P. putida* F39/D, mineral broth, arginine, 28 °C, 48 h, 2 g/L; (b) DMP, *p*‐TsOH, acetone, rt, 30 min; (c) *m*‐CPBA, CH_2_Cl_2_, rt, overnight; (d) ABCC, HBu_3_Sn, THF, reflux; (e) NaN_3_, NH_4_Cl, THF–EtOH–H2O, reflux, 1 h; (f) TMSOTf, CH_2_Cl_2_, −15 °C, 0.5 h; (g) PPh_3_, THF, rt, 24 h, then H_2_O, rt, 4 h; (h) Dowex‐resin (H^+^ form), MeOH and then NH_4_OH 2 M; (i) RuCl_3_–NaIO_4_, AcOEt–CH_3_CN–H_2_O, 0 °C, 3 h; (j) PPh_3_, THF, rt, 24 h, then H_2_O, rt, 24 h; (k) Dowex‐resin (H^+^ form), MeOH and then NH_4_OH 2 M. The percentages in the chemical structures represent the yield of each product formed.

A noteworthy chemoenzymatic approach was reported for synthesizing fagopyritol β‐analogues (Figure 13) with satisfactory yields. Initially, enzymatic catalysis was employed using bromobenzene **86** in a culture of the mutant strain *P. putida* F39/D to introduce chirality and produce *cis*‐cyclohexadienediol **87** with complete regio‐ and stereoselectivity. This homochiral metabolite **87** was then converted into hydroxyazide **88**, serving as a crucial intermediate with a nucleophilic hydroxyl group for glycosylation with derivative **89**, yielding glycoside **90** in excellent yield. To obtain the desired pseudodisaccharide **91**, three final steps were necessary: deacylation, removal of the isopropylidene group, and reduction of the azide group in dimer **90**. To synthesize fagopyritol analog **92**, glycoside **90** was oxidized using catalytic RuCl_3_ and NaIO_4_ as a co‐oxidant. This process resulted in the dihydroxylation of **90**, producing a *syn*‐diol intermediate. Subsequently, after deacylation and removal of the isopropylidene group, the reduction of the azide group yielded fagopyritol analog **92**.^[40]^

### 4.7 A Synthetic Pathway for Mycothiol Through Nickel‐Catalyzed Α‐Glycosylation

**Figure 14:**
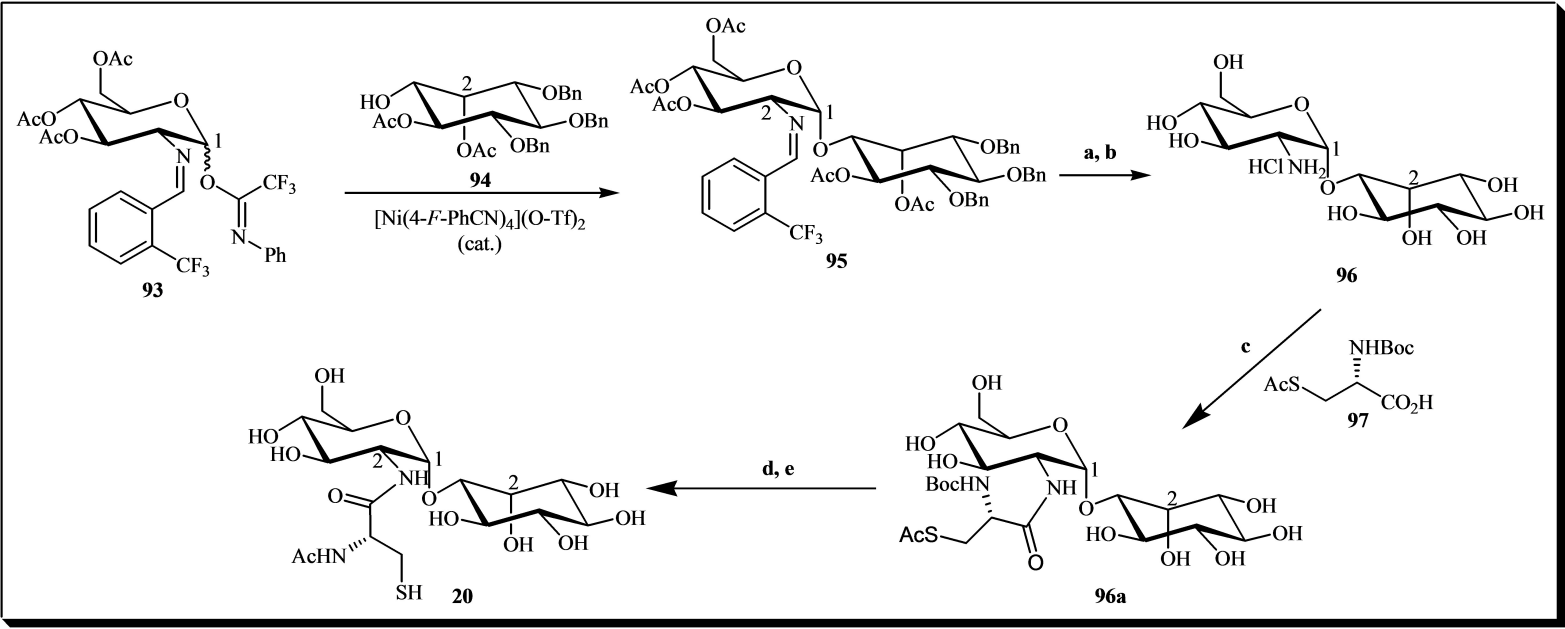
General sequence for the synthesis of mycothiol **20** via nickel‐catalyzed α‐glycosylation. Reagents and conditions: (a) 5 N HCl, acetone, reflux, 5 min; (b) 1. Pd(OH)_2_/C, H_2_, *t*‐BuOH, pH 4 buffer, 2. NaOMe, MeOH; (c) HATU, DIPEA, DMF, 12 h, 78 %; (d) TFA; (e) pyridine.


Mycothiol **20** is a critical low molecular weight thiol essential for defending against various electrophilic agents, such as oxidants, radicals, and drugs. Consequently, several studies have focused on its synthesis.[^36^, ^41^, ^164^] In this regard, a synthetic pathway was developed utilizing nickel‐catalyzed α‐glycosylation. The process employed D‐glucosamine imidate donor **93**, inositol acceptor **94**, and cysteine residue **97** (refer to Figure 14).^[36]^ The synthetic route involved reacting the glycosyl donor **93** with the glycosyl acceptor **94**, yielding α‐pseudodisaccharide **95** in good yields. Subsequently, protective groups were removed to obtain pseudodisaccharide **96**. The coupling of compound **96** with the cysteine derivative **97**, using *O*‐(7‐azabenzotriazol‐1‐yl)‐*N*,*N*,*N′*,*N′*‐tetramethyluronium hexafluorophosphate (HATU) and *N*,*N*‐diisopropylethylamine (DIPEA), yielded the amide **96 a**. Subsequent deprotection of the amine group with trifluoroacetic acid, followed by treatment with pyridine to induce S→N acetyl migration, afforded the target compound **20** in a quantitative yield over two steps.[^164^, ^165^]

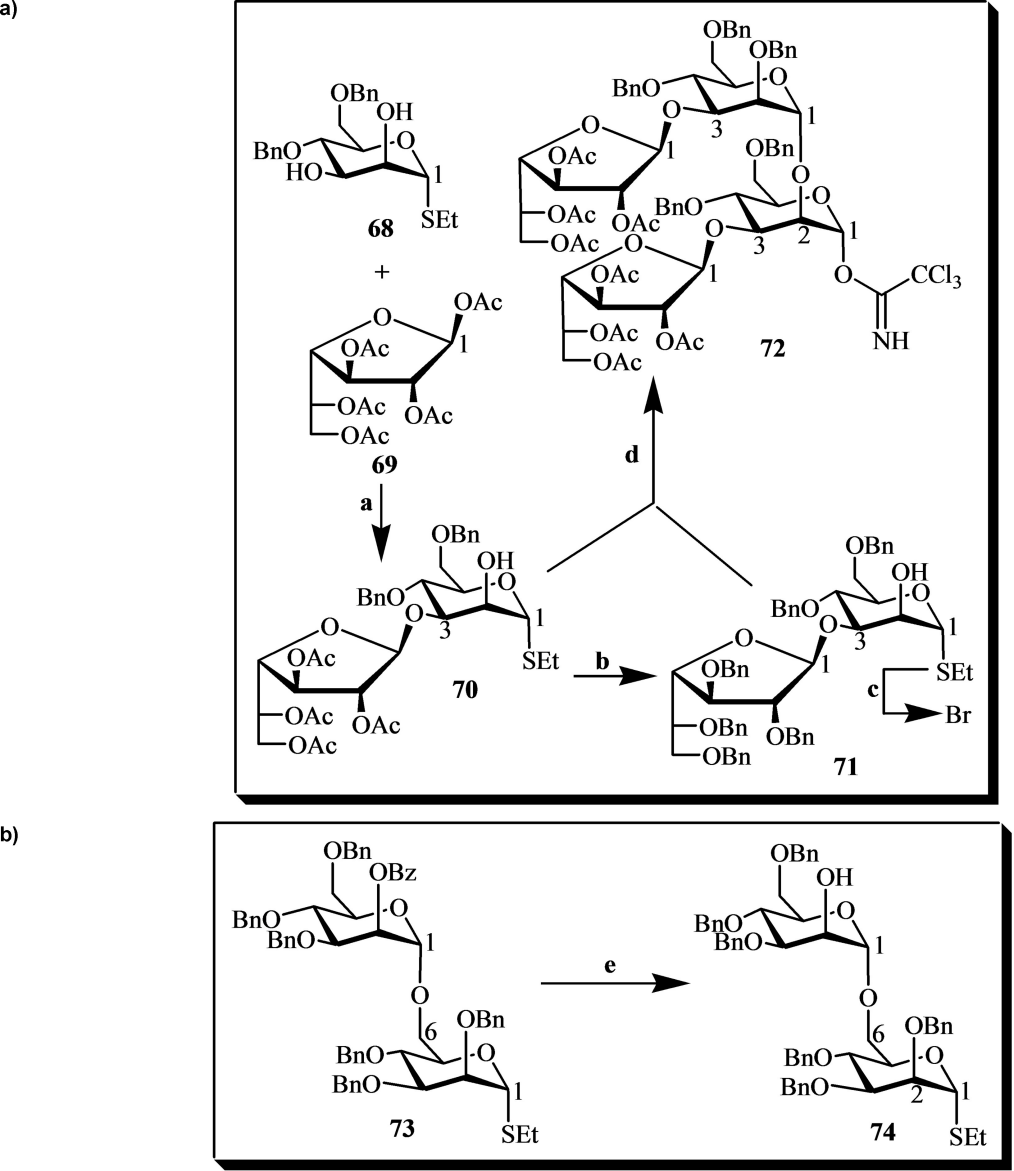


### 4.8 A Synthetic Strategy for Obtaining an Adenophostin A Analog

**Figure 15:**
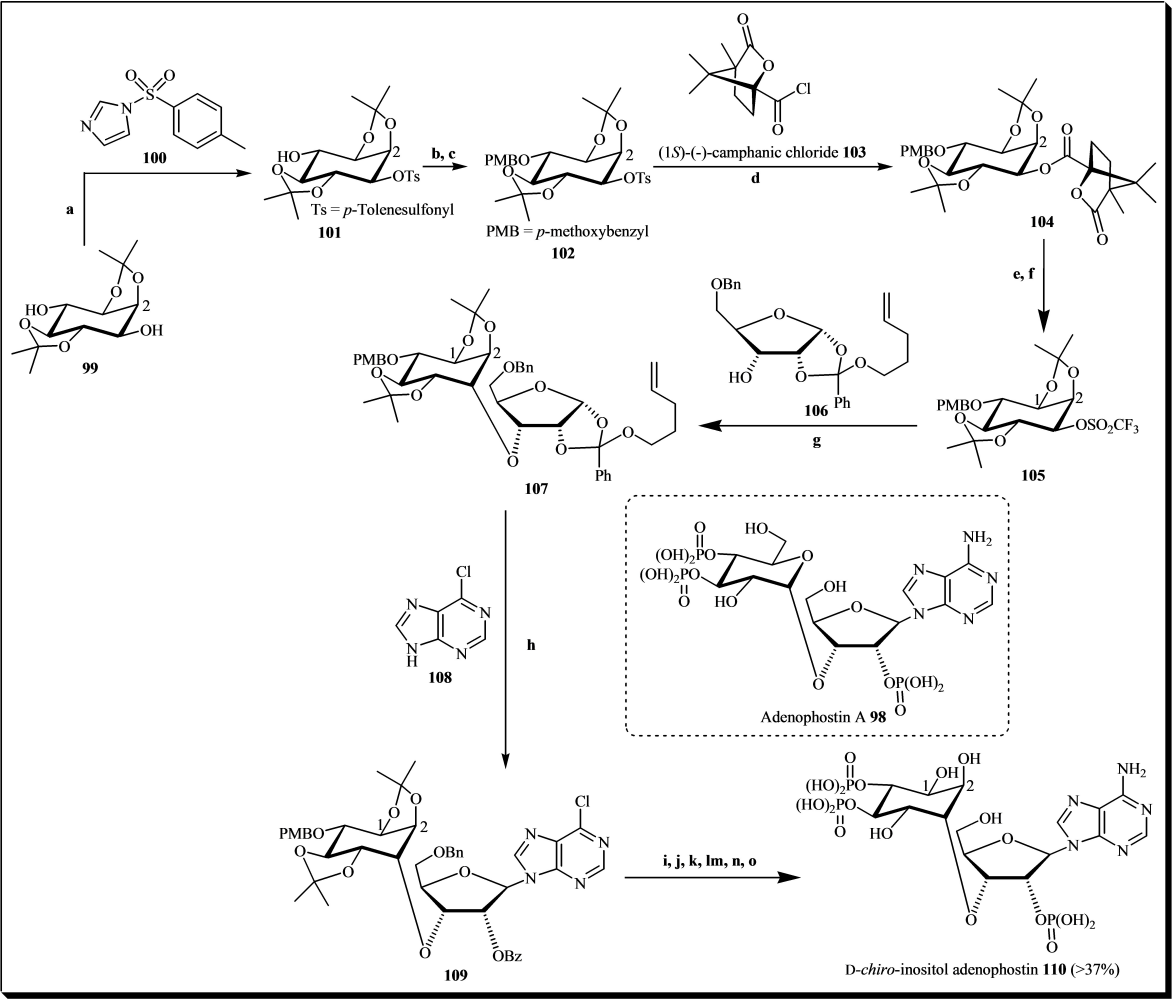
General scheme for the synthesis of D‐*chiro*‐inositol adenophostin **110**. Reagents and conditions: (a) CsF, DMF, rt, 24 h; (b) PMB−Cl, NaH, DMF, 0 °C to rt; (c) Mg, CH_2_Cl_2_/MeOH 1 : 1, rt to reflux, 30 min, then reflux to rt, 1.5 h; (d) DMAP (cat.), CH_2_Cl_2_/pyridine 10 : 1, rt, 18 h; (e) NaOH, MeOH, reflux, 30 min; (f) (SO_2_CF_3_)_2_O, pyridine, CH_2_Cl_2_, 0 °C to rt, 16 h; (g) NaH, HMPA/THF 3 : 1, 0 °C to rt, 18 h; (h) Yb(OSO_2_CF_3_)_3_, NIS, MS 3 Å, MeCN, 0 °C to rt, 16 h; (i) Ethylene glycol, *p*‐TSA (cat.), CH_2_Cl_2_, rt, 20 min; (j) (*n*‐Bu)_2_SnO, MS 3 Å, Soxhlet, MeCN, reflux, 18 h; (k) CsF, BnBr, TBAI (cat.), MS 3 Å, DMF, rt, 72 h; (l) NH_3_, EtOH, 70 °C, 18 h; (m) TFA/CH_2_Cl_2_ 1 : 9, rt, 5 min; (n) 5‐Phenyl‐*1H*‐tetrazole, (BnO)_2_ P−N(*i*‐Pr)_2_, rt, 30 min, then *t*‐BuOOH, rt, 30 min; (o) Cyclohexene (∼600 equiv), Pd(OH)_2_/C (20 %, 5 equiv.), MeOH/H_2_O 9 : 1, 70 °C, 18 h.

Adenophostin A **98** is a potent agonist of the inositol trisphosphate (IP3) receptor, crucial for regulating cytoplasmic free Ca^2+^ levels. Given its importance, a chemical approach was devised to synthesize an analog, D‐*chiro*‐inositol adenophostin **109**. This analog features a nucleoside sugar connected via an axial D‐*chiro*‐inositol 1‐hydroxyl‐adenosine 3′‐ribose ether linkage. The aim was to substitute the α‐glucopyranosyl unit of adenophostin A with a structurally similar inositol unit, functioning as a pseudo‐sugar. The synthesis process involved utilizing the L‐*myo*‐inositol 3‐O‐triflate derivative **104**, the D‐ribose orthoester derivative **105**, and the 6‐chloropurine reactive **107** to produce inositol‐sugar conjugates, followed by deprotection (see Figure 15). The synthesis of the chiral L‐*myo*‐inositol 3‐*O*‐triflate derivative **105** started with racemic 1,2 : 4,5‐di‐*O*‐isopropylidene‐*myo*‐inositol **99**. Key steps in the synthetic approach to generate a triol suitable for phosphorylation included selectively mono‐tosylating **99** using tosyl imidazole **100**, followed by converting the resulting product into a camphanate ester derivative **103**. The addition of the chiral triflate **105** to the alkoxide yielded compound **107**. This was then coupled via its pent‐4‐ene orthoester group with 6‐chloropurine **108** in the presence of an activator under anhydrous conditions, resulting in compound **109** in high yield. Further manipulation of protecting groups, combined *O*‐ and *N*‐phosphorylation, and subsequent removal of all protecting groups in a single step yielded the final product.^[42]^

Since the phosphorylated inositol‐nucleoside hybrid closely resembles adenophostin A, it would be important to determine whether the nature of the phosphorylated glucose moiety is the best mimic of the inositol bisphosphate motif, or if the activity of adenophostin A can be further improved by replacing glucose with a motif more similar to the cyclitol in IP3.

## 5 Enzymatic Synthesis of Glycosyl Inositols

The chemical synthesis of glycosyl inositols presents a significant challenge in organic chemistry, involving long and tedious stages of hydroxyl group protection and deprotection, as illustrated by the examples from the previous section. In contrast, enzymes offer remarkable properties such as chemo‐, regio‐, and stereoselectivity, along with the ability to facilitate reactions under mild conditions without producing toxic waste. Therefore, a biocatalytic approach emerges as a promising alternative to overcome these limitations.[^166^, ^167^, ^168^, ^169^, ^170^] Through the strategic use of enzymes like glycosyltransferases and glycosidases, the glycosylation of diverse chemical compounds has been accomplished using various glycosyl donors.[^166^, ^167^, ^169^]

This review discusses various enzymatic glycosylation strategies utilized in the synthesis of glycosyl inositols. It emphasizes the enzymes employed, such as enzyme extracts from *Sporobolomyces singularis*,[^43^, ^171^] stachyose STS from *Vigna angularis*,^[44]^ β‐galactosidase from *Thermoanaerobacter* sp.,^[45]^ kojibiose phosphorylase from *Thermoanaerobacter brockii*,^[47]^ and CGTase from *Thermoanaerobacter* sp.[^48^, ^49^] For further information, readers are encouraged to refer to the original references cited in this review.

### 5.1 Synthesis of β‐Gluco ‐and β‐Galacto‐Pyranosyl *Myo*‐Inositols Using Growing Cultures and Enzyme Extracts of *Sporobolomyces Singularis*

**Figure 16:**
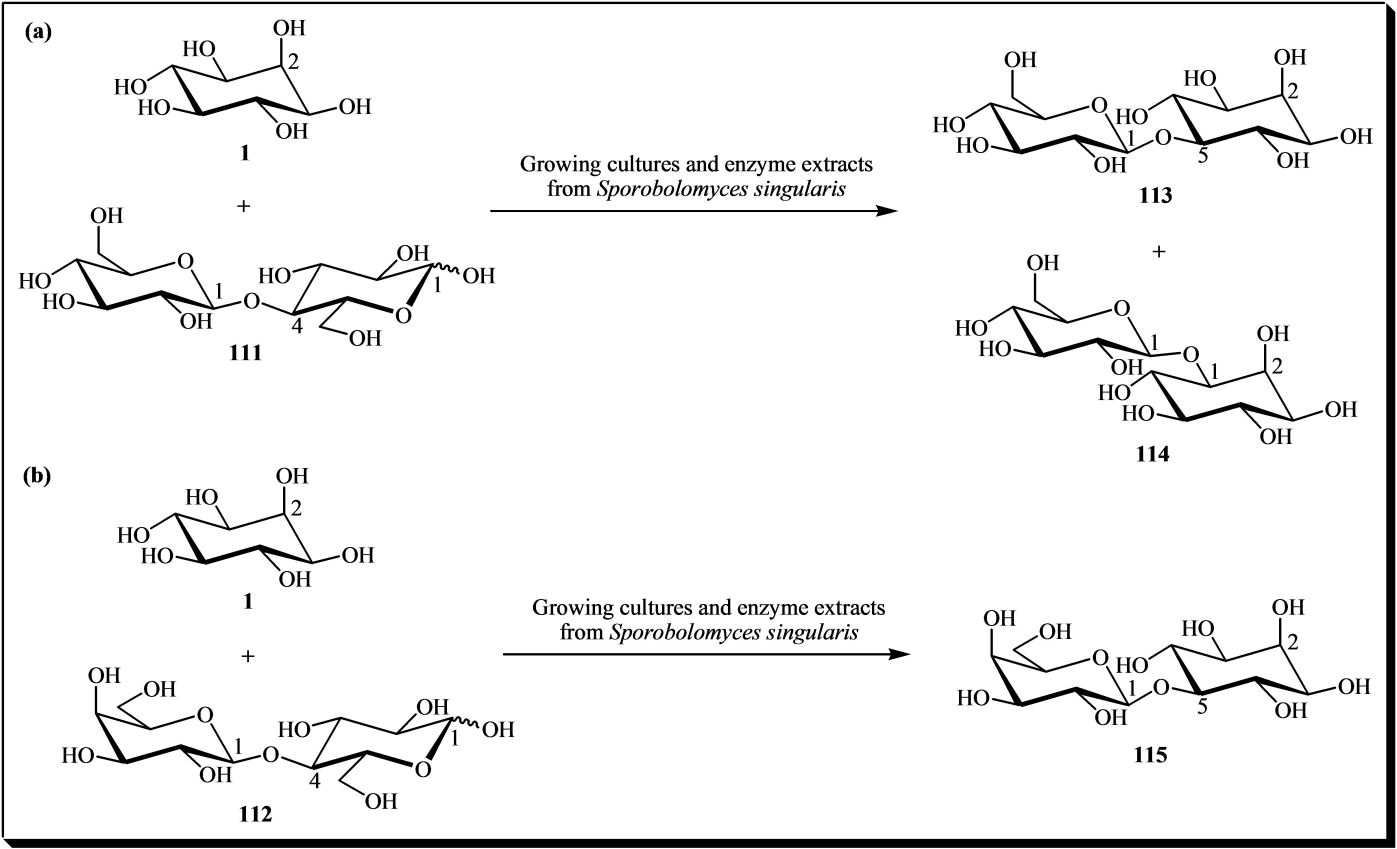
General scheme for the synthesis of some β‐D‐glycosyl *myo*‐inositols using growing cultures and enzyme extracts of *Sporobolomyces singularis*. (a) Using cellobiose as a glucosyl donor; (b) Using lactose as a galactosyl donor. Reagents and conditions: The culture conditions used are described in a previous publication. The reaction contained 5 % cellobiose, 5 % lactose and 2.5 % *myo*‐inositol.

One of the earliest studies on the enzymatic glycosylation of inositols is the research conducted by Gorin et al. (1965). They demonstrated that by using growing cultures and enzyme extracts from *Sporobolomyces singularis*, along with cellobiose **111** or lactose **112** as glycosyl donors, the enzyme could transfer a glucosyl residue to the C1(1 *R*) and C5 positions of *myo*‐inositol **1**, or a galactosyl residue to the C5 position of cyclitol. This process resulted in the formation of transglycosylation products **113**–**115**. They also observed that the configuration of hydroxyl groups adjacent to the substituted position is the same in these products as in the corresponding pyranoid derivatives (Figure 16).[^43^, ^171^] In reactions utilizing cellobiose as a glycosyl donor, pseudo disaccharide **113** constituted 38 % of the glucosyl inositol fraction, whereas product **115** was isolated with a yield of 23 %.^[43]^

### 5.2 Synthesis of β‐Galactosyl Inositols and β‐Galactosyl Inositol *O*‐Methyl‐Ethers with Stachyose STS from *V. Angularis*

Various isomeric inositols and inositol *O*‐methyl ethers were evaluated as galactosyl acceptors in reactions involving stachyose STS (EC 2.4.1.67) from *Vigna angularis*, with galactinol as the galactosyl donor. Optimal conditions of the enzyme revealed that *myo*‐inositol **1** was the most efficient acceptor, followed by *epi*‐inositol **8**, *scyllo*‐inositol **9**, bornesitol **10**, sequoyitol **12**, D‐pinitol **14**, and 1‐*O*‐methyl‐*scyllo*‐inositol **116**, resulting in the synthesis of galactosylcyclitols. However, the specific glycosylation positions were not specified by the authors.^[44]^

### 5.3 Synthesis of Galactosylated Inositols Using β‐Galactosidase from *Thermoanaerobacter* sp.

**Figure 17:**
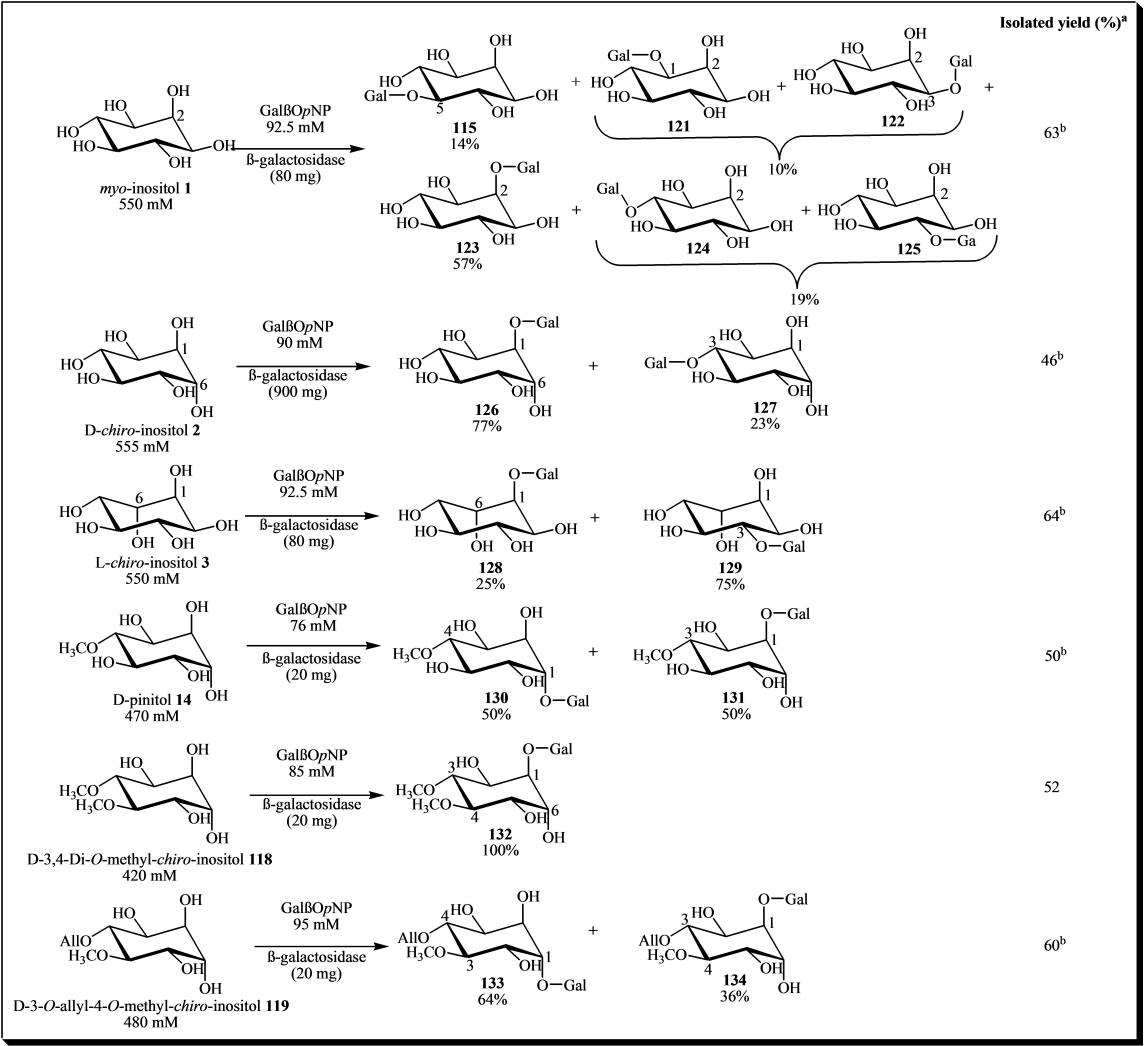
Products from galactosylation of various inositols catalyzed by β‐galactosidase isolated from *Thermoanaerobacter* sp. TP6‐B. Reagents and conditions: *p*‐nitrophenyl β‐D‐galactopyranoside (GalβO*p*NP) and inositols were incubated in a phosphate buffer (pH 7.0, 0.1 M NaH_2_PO_4_) with the β‐galactosidase from TP6‐B1 at 37 °C during 72 h. *The percentages in the chemical structures represent the proportion of each product formed. ^a^Yields based on a limiting reagent, that is GalβO*p*NP. ^b^Combined yield. Gal=Galactose residue.

The β‐galactosidase obtained from *Thermoanaerobacter* sp. strain TP6‐B1 exhibits a preference for hydrolyzing β‐linked galactopyranosides, such as *o*‐ and *p*‐nitrophenyl β‐D‐galactopyranoside and lactose. However, in a study investigating its transglycosylation capabilities, Hart et al. (2004b) reported the synthesis of multiple β‐D‐galactosyl‐inositols (compounds **115**–**134**) with yields ranging from 46 % to 64 % (see Figure 17). A structural analysis of the products from these reactions provided some insight into the hydroxyl group configurations that are key to recognizing the acceptor as a substrate in the enzyme's active site.^[45]^

In separate studies, D‐*chiro*‐inositol **2** and D‐pinitol **14** were successfully galactosylated using a β‐galactosidase sourced from *Bacillus circulans*, yielding mono‐ and digalactosylated inositols with yields reaching up to 45 %.^[46]^

### 5.4 Syntheses of Glycosyl *Myo*‐Inositols by a Kojibiose Phosphorylase from *Thermoanaerobacter Brockii*

**Figure 18:**
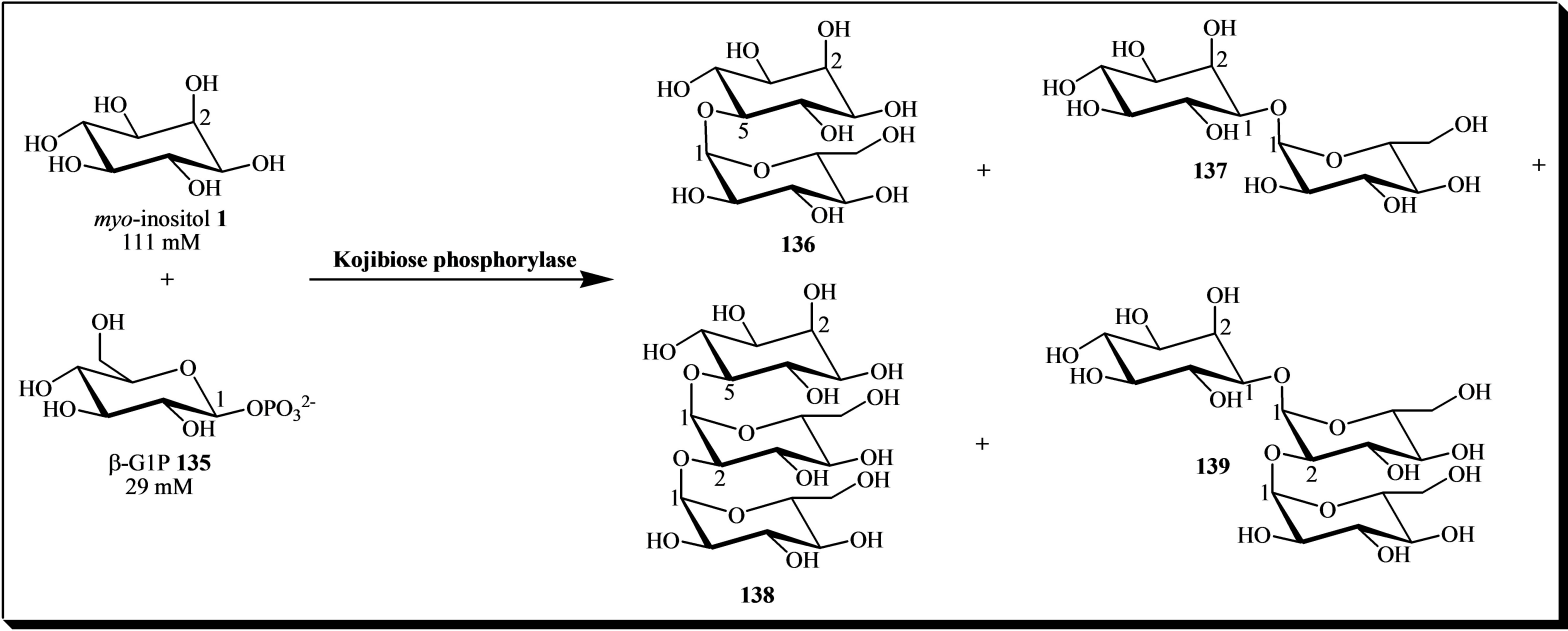
General scheme for the synthesis of α‐D‐glucosyl *myo*‐inositols catalyzed by kojibiose phosphorylase. The reaction mixture (2.0 ml) included an enzyme solution (0.2 ml), *myo*‐inositol, and β‐G1P. The reaction was conducted in a 50 mM acetate buffer (pH 5.5) at 60 °C for 30 minutes.

In addition to glycosidases, inositol glycosylation has been achieved using other enzymes, such as kojibiose phosphorylase from *Thermoanaerobacter brockii*. This enzyme was used as a biocatalyst in the synthesis of four glucosyl‐*myo*‐inositols **136**–**139**, utilizing β‐D‐glucose 1‐phosphate (β‐G1P) **135** as the glucosyl donor (see Figure 18). Structural analysis of the glycosylation products showed that *myo*‐inositol **1** shares a common structure with three equatorially positioned hydroxyl groups corresponding to those of the glucose molecule at positions 2, 3, and 4. These hydroxyl groups constitute the substrate recognition site of kojibiose phosphorylase from *Thermoanaerobacter brockii*.^[47]^

### 5.5 Regioselective α‐D‐Glucosylation of Inositols Catalyzed by CGTase from *Thermoanaerobacter* sp.

**Figure 19:**
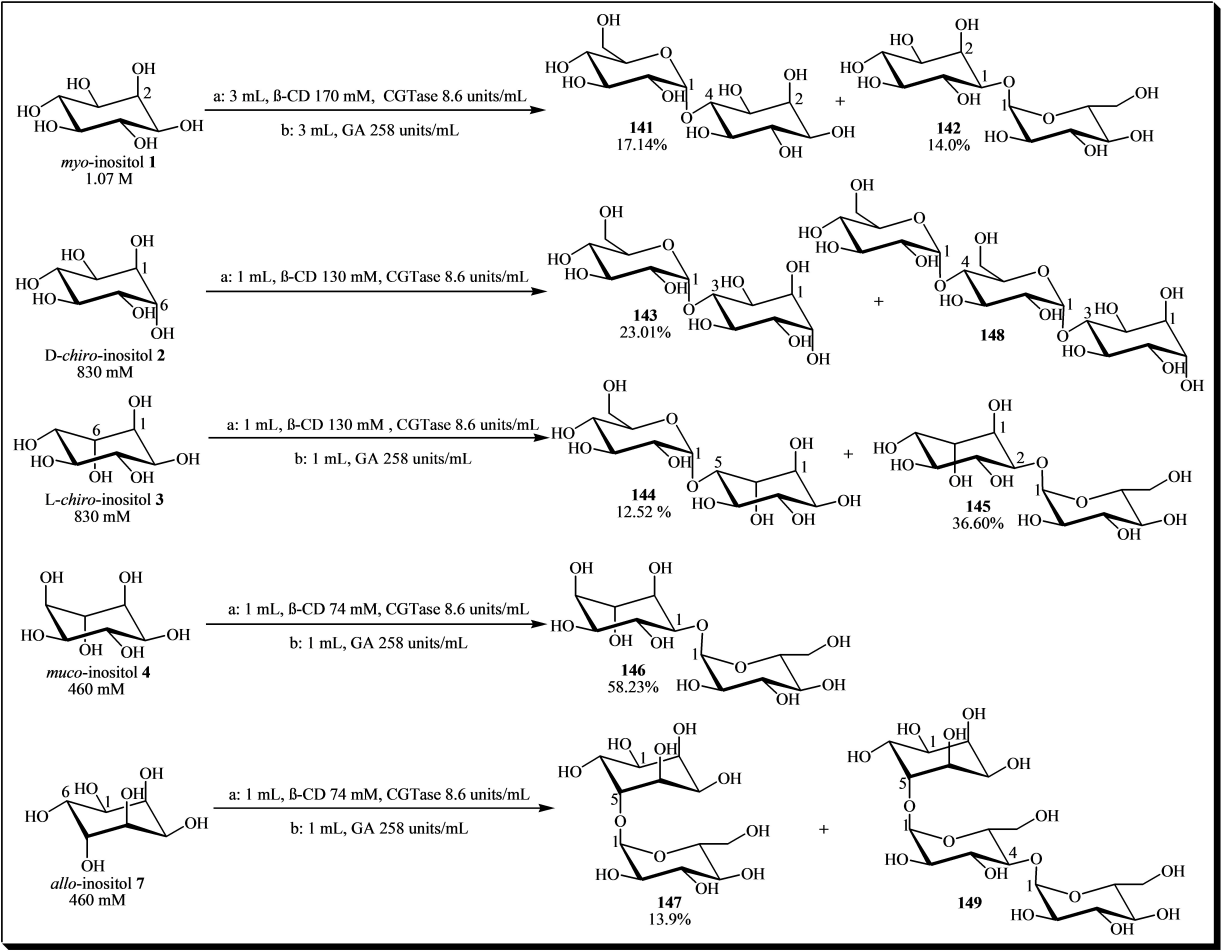
Combined enzymatic transglycosylation and hydrolysis strategy for the synthesis of α‐D‐glucosyl‐inositols: (a) Transglucosylation using CGTase from *Thermoanaerobacter* sp., where inositol and β‐CD were incubated in a phosphate buffer (pH 6.0, 50 mM Na_2_HPO_4_). (b) Selective hydrolysis of the poly‐glucosylated products using *Aspergillus niger* glucoamylase (GA) as biocatalysts. The percentages in the chemical structures indicate the yield of each product formed. Both reactions were conducted at 50 °C for 24 hours.

In a subsequent study, seven α‐D‐monoglucosyl‐inositols (**141**–**149**) were synthesized using a combined enzymatic transglycosylation and hydrolysis strategy. This approach involved CGTase from *Thermoanaerobacter* sp. as the biocatalyst, β‐cyclodextrin (β‐CD) **140** as the glucosyl donor, and several inositols as glycosyl acceptors, followed by hydrolysis with *Aspergillus niger* glucoamylase (see Figure 19).[^48^, ^49^]

When *myo*‐inositol **1** was used as an acceptor, two regioisomeric products, **141** and **142**, were produced. Both isomers exhibited comparable *in vivo* anti‐inflammatory activity to corticosterone, as demonstrated in mouse ear edema induced by 12‐*O*‐tetradecanoylphorbol‐13‐acetate and in rat hind paw edema induced by carrageenan.^[49]^

Enzymatic glycosylation experiments using D‐*chiro*‐inositol **2**, *muco*‐inositol **4**, and *allo*‐inositol **7** as glucosyl acceptors showed the formation of a single monoglucosylated product in each reaction. In contrast, reactions involving L‐*chiro*‐inositol **3** resulted in the formation of two pseudo‐disaccharide products. Additionally, reactions with D‐*chiro*‐inositol **2** and *allo*‐inositol **7** yielded two pseudo‐trisaccharide products (compounds **148** and **149**).^[48]^ The enzymatic regioselectivity was attributed to the enzyme's preference for hydroxyl groups in equatorial positions, similar to the 1‐OH and/or 4‐OH positions found in β‐D‐glucose, which is a natural acceptor for CGTase.[^48^, ^49^]

Another successful approach for synthesizing glucosylated *myo*‐inositol was reported by Sato et al. (1992). They utilized *myo*‐inositol **1** as the acceptor and β‐CD **140** as the glucosyl donor in a transglycosylation reaction catalyzed by CGTase from *Bacillus obhensis*. The study resulted in the isolation and identification of the monoglucosyl *myo*‐inositol regioisomers **136** and **142**. Moreover, the glycosylated products demonstrated efficacy as prebiotics. Specifically, maltosyl‐*myo*‐inositol exhibited the most potent growth stimulation effect on intestinal Bifidobacterium.^[50]^ Additional studies documented the enzymatic production of glucosyl‐*myo*‐inositols using CGTase from *Bacillus macerans* and α‐amylase from *Bacillus licheniformis*, achieving yields of up to 56.4 % and 2.2 %, respectively. However, this report did not provide specific details regarding the glycosylation sites on the cyclitol.^[172]^

## 6 Conclusions

This review provides a comprehensive exploration of glycosyl inositols, focusing on their biological properties, synthetic routes, and potential applications. Glycosyl inositols are highlighted as a diverse class of compounds with significant biological relevance, found in natural sources and playing crucial roles in signal transduction, metabolic regulation, and stress response across plant and microbial systems.

Synthesizing glycosyl inositols presents challenges due to their complex structures, requiring precise control over stereochemistry and regiochemistry. Both chemical and enzymatic methods are explored as viable approaches to overcome these challenges, aiming to facilitate scalable production for diverse industrial and therapeutic uses, despite their limited natural availability.

Overall, this review underscores the evolving study of glycosyl inositols, driven by their intricate chemistry and promising biological activities. This research opens new avenues for exploration in medicine, agriculture, and biotechnology, indicating significant potential for future application.

## 7 Declaration of Interest Statement

The authors declare no conflict of interest.

## 8 Funding

This research was funded by UNAM DGAPA‐PAPIIT IA209220 and the Consejo Nacional de Humanidades, Ciencias y Tecnologías (Grants CB2014‐01 240801 and LN 315896). Alfonso Miranda Molina expresses gratitude to SECIHTI for the support received, CVU number 164831, under the “Convocatoria 2022(1) Estancias Posdoctorales por México – Modalidad Académica.”

## Conflict of Interests

The authors declare no conflict of interest.

9

## Biographical Information

*Dr. Alfonso Miranda Molina is a biotechnologist with expertise in chemistry and food sciences. He earned a Ph.D. in chemistry from the Universidad Autónoma del Estado de Morelos and completed postdoctoral research at the Instituto de Biotecnología‐UNAM. His work focuses on enzymatic synthesis, polysaccharides, and biocatalysis, driving advancements in both industry and academia. As a faculty member at Tecnológico de Monterrey, he has taught various chemistry courses. A prolific researcher, he has published extensively in high‐impact journals, contributing to the understanding of macromolecule structuring and biotechnological applications. His research continues to foster innovation in chemical and biotechnological processes*.

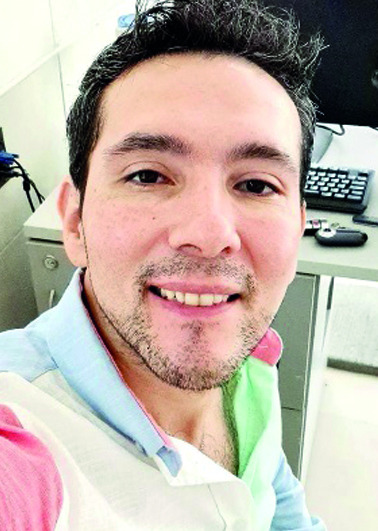

## Biographical Information

*Dra. Laura Alvarez Berber is a professor‐researcher at the Chemical Research Center of the Autonomous University of the State of Morelos. She teaches diverse chemical curses at the Bachelor and postgraduate degrees. Her research interest is related with the isolation and characterization of the molecular chemical structure of the constituents responsible for the pharmacological activity present in Mexican medicinal plants. Among the lines of research developed, the obtaining of new secondary metabolites from plants and studies of structure‐activity relationship of active natural products and structural studies of the ligand‐receptor interaction associated with cancer and inflammation stand out*.

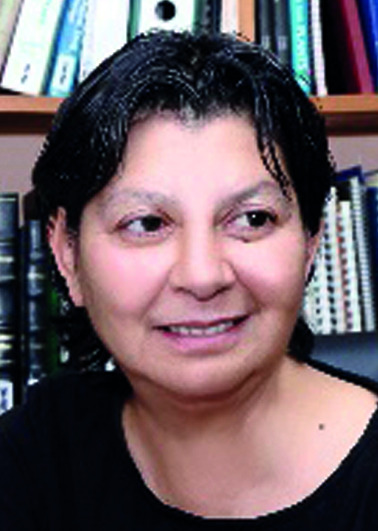

## Biographical Information

*Mayra Antúnez is a young research associate from SECIHITI at Autonomous University of the State of Morelos. She holds a Ph.D. in Science (Chemistry). Her research focusses on bioactive compounds exhibiting anticancer, anti‐inflammatory, antioxidant, and prebiotic activities, derived from functional foods and medicinal plants. Her expertise includes the purification and structural elucidation of secondary metabolites, as well as molecular recognition through Nuclear Magnetic Resonance (NMR). She has conducted research stays at the Center for Biological Research in Madrid, Spain, specializing in the NMR Molecular Recognition Laboratory and in the Structure and Function of the Cytoskeleton, Pharmacology, and Vaccines Laboratory*.

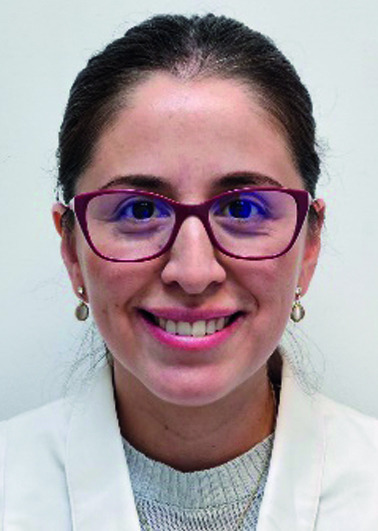

## Biographical Information

*Benjamín Velasco Bejarano obtained his PhD in Medicinal Chemistry from Higer School of Medicine of the National Polytechnic Institute in Mexico in 2006. He obtained a Posdoctoral Fellow in 2008 at the Chemical Research Center of the Autonomous University of the State of Morelos. He is a professor‐researcher at the Faculty of Higher Studies Cuautitlan of the National Autonomous University of México. The primary interest of his group revolve around synthesis of molecules with biological activity using green chemistry protocols and biocatalytic as well as enzymology procedures*.

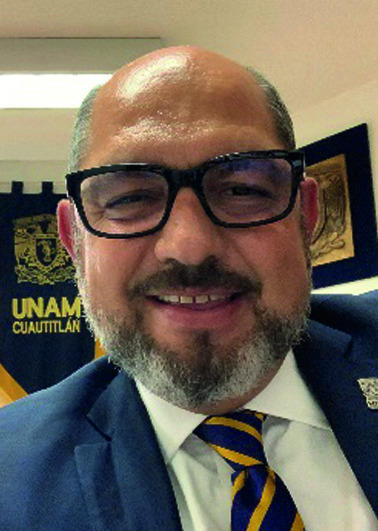

## Data Availability

The data that support the findings of this study are available on request from the corresponding author. The data are not publicly available due to privacy or ethical restrictions.
